# Chromosome-scale genome sequence of *Suaeda glauca* sheds light on salt stress tolerance in halophytes

**DOI:** 10.1093/hr/uhad161

**Published:** 2023-08-10

**Authors:** Yan Cheng, Jin Sun, Mengwei Jiang, Ziqiang Luo, Yu Wang, Yanhui Liu, Weiming Li, Bing Hu, Chunxing Dong, Kangzhuo Ye, Zixian Li, Fang Deng, Lulu Wang, Ling Cao, Shijiang Cao, Chenglang Pan, Ping Zheng, Sheng Wang, Mohammad Aslam, Hong Wang, Yuan Qin

**Affiliations:** State Key Laboratory of Ecological Pest Control for Fujian and Taiwan Crops, College of Plant Protection, Fujian Provincial Key Laboratory of Haixia Applied Plant Systems Biology, Center for Genomics and Biotechnology, College of Life Science, Fujian Agriculture and Forestry University, Fuzhou 350002, China; Pingtan Institute of Science and Technology, Fujian Agriculture and Forestry University, Fuzhou 350400, China; Department of Biochemistry, Microbiology and Immunology, University of Saskatchewan, Saskatoon, SK S7N 5E5, Canada; State Key Laboratory of Ecological Pest Control for Fujian and Taiwan Crops, College of Plant Protection, Fujian Provincial Key Laboratory of Haixia Applied Plant Systems Biology, Center for Genomics and Biotechnology, College of Life Science, Fujian Agriculture and Forestry University, Fuzhou 350002, China; Pingtan Institute of Science and Technology, Fujian Agriculture and Forestry University, Fuzhou 350400, China; College of Agriculture, Fujian Agriculture and Forestry University, Fuzhou 350002, China; State Key Laboratory of Ecological Pest Control for Fujian and Taiwan Crops, College of Plant Protection, Fujian Provincial Key Laboratory of Haixia Applied Plant Systems Biology, Center for Genomics and Biotechnology, College of Life Science, Fujian Agriculture and Forestry University, Fuzhou 350002, China; State Key Laboratory of Ecological Pest Control for Fujian and Taiwan Crops, College of Plant Protection, Fujian Provincial Key Laboratory of Haixia Applied Plant Systems Biology, Center for Genomics and Biotechnology, College of Life Science, Fujian Agriculture and Forestry University, Fuzhou 350002, China; College of Agriculture, Fujian Agriculture and Forestry University, Fuzhou 350002, China; State Key Laboratory of Ecological Pest Control for Fujian and Taiwan Crops, College of Plant Protection, Fujian Provincial Key Laboratory of Haixia Applied Plant Systems Biology, Center for Genomics and Biotechnology, College of Life Science, Fujian Agriculture and Forestry University, Fuzhou 350002, China; College of Agriculture, Fujian Agriculture and Forestry University, Fuzhou 350002, China; State Key Laboratory of Ecological Pest Control for Fujian and Taiwan Crops, College of Plant Protection, Fujian Provincial Key Laboratory of Haixia Applied Plant Systems Biology, Center for Genomics and Biotechnology, College of Life Science, Fujian Agriculture and Forestry University, Fuzhou 350002, China; State Key Laboratory of Ecological Pest Control for Fujian and Taiwan Crops, College of Plant Protection, Fujian Provincial Key Laboratory of Haixia Applied Plant Systems Biology, Center for Genomics and Biotechnology, College of Life Science, Fujian Agriculture and Forestry University, Fuzhou 350002, China; Pingtan Institute of Science and Technology, Fujian Agriculture and Forestry University, Fuzhou 350400, China; State Key Laboratory of Ecological Pest Control for Fujian and Taiwan Crops, College of Plant Protection, Fujian Provincial Key Laboratory of Haixia Applied Plant Systems Biology, Center for Genomics and Biotechnology, College of Life Science, Fujian Agriculture and Forestry University, Fuzhou 350002, China; College of Agriculture, Fujian Agriculture and Forestry University, Fuzhou 350002, China; State Key Laboratory of Ecological Pest Control for Fujian and Taiwan Crops, College of Plant Protection, Fujian Provincial Key Laboratory of Haixia Applied Plant Systems Biology, Center for Genomics and Biotechnology, College of Life Science, Fujian Agriculture and Forestry University, Fuzhou 350002, China; Pingtan Institute of Science and Technology, Fujian Agriculture and Forestry University, Fuzhou 350400, China; State Key Laboratory of Ecological Pest Control for Fujian and Taiwan Crops, College of Plant Protection, Fujian Provincial Key Laboratory of Haixia Applied Plant Systems Biology, Center for Genomics and Biotechnology, College of Life Science, Fujian Agriculture and Forestry University, Fuzhou 350002, China; Pingtan Institute of Science and Technology, Fujian Agriculture and Forestry University, Fuzhou 350400, China; State Key Laboratory of Ecological Pest Control for Fujian and Taiwan Crops, College of Plant Protection, Fujian Provincial Key Laboratory of Haixia Applied Plant Systems Biology, Center for Genomics and Biotechnology, College of Life Science, Fujian Agriculture and Forestry University, Fuzhou 350002, China; State Key Laboratory of Ecological Pest Control for Fujian and Taiwan Crops, College of Plant Protection, Fujian Provincial Key Laboratory of Haixia Applied Plant Systems Biology, Center for Genomics and Biotechnology, College of Life Science, Fujian Agriculture and Forestry University, Fuzhou 350002, China; State Key Laboratory of Ecological Pest Control for Fujian and Taiwan Crops, College of Plant Protection, Fujian Provincial Key Laboratory of Haixia Applied Plant Systems Biology, Center for Genomics and Biotechnology, College of Life Science, Fujian Agriculture and Forestry University, Fuzhou 350002, China; Department of Biochemistry, Microbiology and Immunology, University of Saskatchewan, Saskatoon, SK S7N 5E5, Canada; College of Forestry, Fujian Agriculture and Forestry University, Fuzhou 350002, China; Fujian Key Laboratory on Conservation and Sustainable Utilization of Marine Biodiversity, Fuzhou Institute of Oceanography, Minjiang University, Fuzhou 350108, China; State Key Laboratory of Ecological Pest Control for Fujian and Taiwan Crops, College of Plant Protection, Fujian Provincial Key Laboratory of Haixia Applied Plant Systems Biology, Center for Genomics and Biotechnology, College of Life Science, Fujian Agriculture and Forestry University, Fuzhou 350002, China; Pingtan Institute of Science and Technology, Fujian Agriculture and Forestry University, Fuzhou 350400, China; Department of Biochemistry, Microbiology and Immunology, University of Saskatchewan, Saskatoon, SK S7N 5E5, Canada; State Key Laboratory of Ecological Pest Control for Fujian and Taiwan Crops, College of Plant Protection, Fujian Provincial Key Laboratory of Haixia Applied Plant Systems Biology, Center for Genomics and Biotechnology, College of Life Science, Fujian Agriculture and Forestry University, Fuzhou 350002, China; Pingtan Institute of Science and Technology, Fujian Agriculture and Forestry University, Fuzhou 350400, China; Department of Biochemistry, Microbiology and Immunology, University of Saskatchewan, Saskatoon, SK S7N 5E5, Canada; State Key Laboratory of Ecological Pest Control for Fujian and Taiwan Crops, College of Plant Protection, Fujian Provincial Key Laboratory of Haixia Applied Plant Systems Biology, Center for Genomics and Biotechnology, College of Life Science, Fujian Agriculture and Forestry University, Fuzhou 350002, China; Pingtan Institute of Science and Technology, Fujian Agriculture and Forestry University, Fuzhou 350400, China

## Abstract

Soil salinity is a growing concern for global crop production and the sustainable development of humanity. Therefore, it is crucial to comprehend salt tolerance mechanisms and identify salt-tolerance genes to enhance crop tolerance to salt stress. *Suaeda glauca*, a halophyte species well adapted to the seawater environment, possesses a unique ability to absorb and retain high salt concentrations within its cells, particularly in its leaves, suggesting the presence of a distinct mechanism for salt tolerance. In this study, we performed *de novo* sequencing of * the S. glauca* genome. The genome has a size of 1.02 Gb (consisting of two sets of haplotypes) and contains 54 761 annotated genes, including alleles and repeats. Comparative genomic analysis revealed a strong synteny between the genomes of *S. glauca* and *Beta vulgaris*. Of the *S. glauca* genome, 70.56% comprises repeat sequences, with retroelements being the most abundant. Leveraging the allele-aware assembly of the *S. glauca* genome, we investigated genome-wide allele-specific expression in the analyzed samples. The results indicated that the diversity in promoter sequences might contribute to consistent allele-specific expression. Moreover, a systematic analysis of the ABCE gene families shed light on the formation of *S. glauca*’s flower morphology, suggesting that dysfunction of A-class genes is responsible for the absence of petals in *S. glauca*. Gene family expansion analysis demonstrated significant enrichment of Gene Ontology (GO) terms associated with DNA repair, chromosome stability, DNA demethylation, cation binding, and red/far-red light signaling pathways in the co-expanded gene families of *S. glauca* and *S. aralocaspica*, in comparison with glycophytic species within the chenopodium family. Time-course transcriptome analysis under salt treatments revealed detailed responses of *S. glauca* to salt tolerance, and the enrichment of the transition-upregulated genes in the leaves associated with DNA repair and chromosome stability, lipid biosynthetic process, and isoprenoid metabolic process. Additionally, genome-wide analysis of transcription factors indicated a significant expansion of *FAR1* gene family. However, further investigation is needed to determine the exact role of the *FAR1* gene family in salt tolerance in *S. glauca*.

## Introduction

Soil salinity is a significant issue that impacts agricultural development globally. Currently, it is estimated that the global area of saline–alkali land has reached 9.5 × 10^8^ hm^2^, with ~20% of arable land and nearly half of the irrigated land affected by salinity. Furthermore, saline–alkali land is increasing at a rate of 1 million to 1.5 million square hectometers per year, and by 2050 more than 50% of cultivated land is expected to be salinized [1]. Land with salt content >0.6% is considered heavily salinized [2], which is a significant limitation for local agricultural development, as most crops cannot grow on this type of land [3]. Therefore, it is crucial to uncover genetic resources and mechanisms that can breed more adaptable crop varieties to ensure global food security [4].

**Figure 1:**
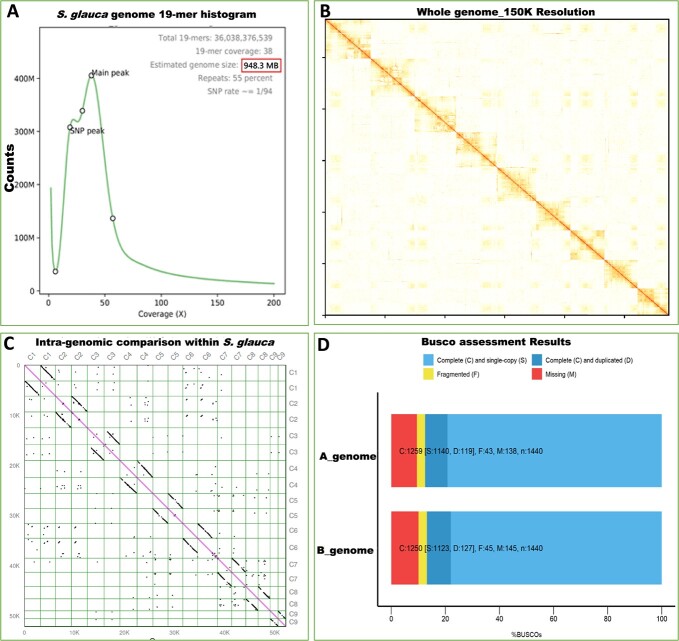
Genome size estimation and *de novo* genome assembly of *S. glauca*. (**A**) Genome survey results for *S. glauca*. (**B**) Hi-C heat map of chromosomal loci interactions throughout the genome, with 150 kb resolution. (**C**) Intra-genomic comparison of *S. glauca*, showing the consistency of two sets of haploid chromosomes**.** (**D**) BUSCO assessment results for two sets of haploid genomes.

**Figure 2:**
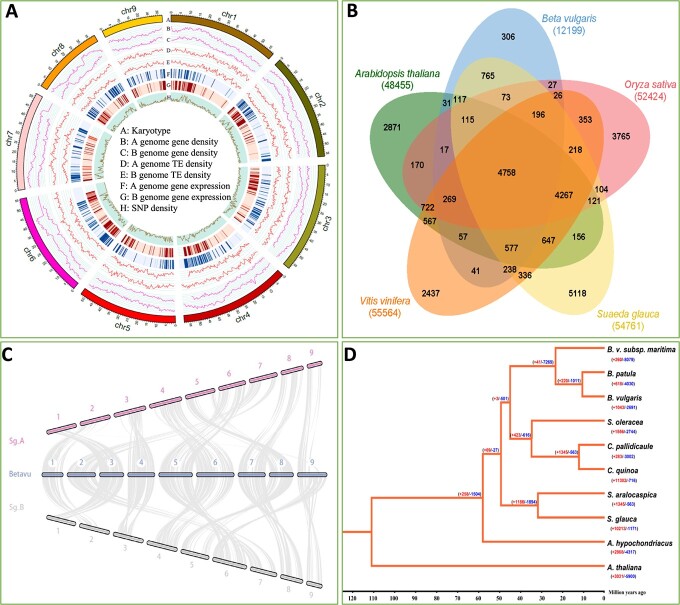
Annotation and phylogenetic analysis of the *S. glauca* genome. (**A**) Landscape of *S. glauca* genome, showing karyotype, A genome gene density, B genome gene density, A genome TE density, B genome TE density, A genome gene expression, B genome gene expression, and SNP density from outer (A) to inter (H) circular diagrams. Resolution = 500 K. (**B**) Orthologous gene families among *S. glauca*, *A. thaliana*, *B. vulgaris*, *O. sativa*, and *V. vinifera*. (**C**) Synteny between two sets of *S. glauca* (Sg) haploid genomes and the *B. vulgaris* (Betavu) genome. (**D**) Phylogenetic tree displaying the genetic relationship between *S. glauca* and other species.

Halophytes are plants that can tolerate growing in soil with a salt content of >200 mM NaCl [5]. *Suaeda glauca*, an annual halophyte, is a dominant species on saline lands worldwide [6]. It belongs to the Amaranthaceae family, which is known for its salt resistance and contains several crops and wild cultivars with excellent salt tolerance [7–9]. Recently, the genomes of some Amaranthaceae family members, including sugar beet (*Beta vulgaris*), spinach (*Spinacia oleracea*), quinoa (*Chenopodium quinoa*), and *Suaeda aralocaspica*, have been deciphered, and the mechanisms of their salt tolerance have been investigated to some extent [10–14]. *Suaeda glauca* can tolerate salinity up to 600 mM NaCl and can absorb high concentrations of salt and retain them within its cells, particularly its leaf cells, indicating a unique mechanism underlying its salt tolerance. It is also used as a pioneer plant for soil salination remediation, and its seedlings are edible, while seeds have the potential for oil extraction [15]. Previous reports suggest that the chromosome number of *S. glauca* is 18 (2*n*), and the genome size is ~500 Mb, representing the smallest genome in this family [16]. However, research to understand the biological characteristics of *S. glauca* on the genomic scale has not been conducted. The deciphering of its genome would have significant implications for investigating the plant’s tolerance of high salt concentrations.

Halophytes have developed various mechanisms to cope with high concentrations of salt stress, even within the same family. For instance, quinoa possesses epidermal bladder cells (EBCs), which are responsible for secreting salt out of the plant, resulting in its high salt-stress potential [13]. On the other hand, *S. glauca* does not have salt glands or bladders; instead, its leaves are succulent, and specialized parenchymatous tissue stores salt and water in giant vacuoles [17], suggesting that there might be a different salt-tolerance mechanism in *S. glauca*. Under salt stress, *Suaeda* species protect their cells by increasing osmoregulatory substances and enhancing their antioxidant system [17, 18]. *Carpobrotus rossii*, on the other hand, stores salt ions in vacuoles to prevent leakage and this is crucial for its ability to deposit Na^+^, although the molecular regulation behind it is still not well understood [19, 20]. Salt stress induces the production of reactive oxygen species (ROS) and interferes with the function of K^+^-containing enzymes, resulting in cellular damage, including DNA damage and abnormal chromosomes, which is a less studied aspect in halophytes [21, 22].

DNA integrity and chromosome stability are essential for plant survival, but high salinity can induce DNA lesions and inhibit DNA repair. In *Arabidopsis*, high salt levels have been shown to double the number of mutations compared with the control [23, 24]. However, a previous study demonstrated that plants reverted to their morphology under salt stress after knocking out a gene responsible for recognizing DNA damage and initiating repair processes, indicating a complex relationship between salt stress and genetic damage repair [25]. *S. glauca* maintains normal growth and reproduction despite continuous pressure generated by salt storage in its vacuoles. Further research is necessary to understand how *S. glauca* overcomes the risk of salt-induced chromosomal abnormalities during the seed germination period [26]. Genome analysis of *S. glauca* will provide valuable insights into these processes and eventually help in the development of molecular-level breeding strategies for creating salt-tolerant crops.

## Results

### *De novo* sequencing of the chromosome-level haplotypic genome of *S. glauca*

The analysis of 50.2 Gb of data generated from the HiSeq 2500 PE150 platform was used to obtain more detailed information about the genome of *S. glauca*. We performed *k*-mer analysis to estimate the genome size and analyze heterozygosity. The total number of 19-mers was 36 038 376 539, and the 19-mer frequency distribution curve exhibited two peaks at a depth of 25 and 48, with an average *k*-mer coverage of 38. The genome size of *S. glauca* was estimated to be 948 378 330 bp using the formula of genome size = total *k*-mer number/peak depth. The first peak at approximately half of the main peak depth indicated a high level of heterozygosity for this species. Simulation analysis using the *Arabidopsis* genome revealed a 1% SNP (heterozygosity) rate (Fig. 1A). The content of repetitive sequences was estimated to be 55%.

The PacBio RSII platform was used to generate the circular consensus sequencing (CCS) reads of the *S. glauca* genome, and ~100 Gb subreads were obtained for contig assembly. A total of 15 340 contigs with an N50 of 0.6 M and a total length of 1 027 094 429 bp were obtained (Supplementary Data Table S1). We previously reported that the chromosome number of *S. glauca* is 2*n* = 18. To anchor the contigs to chromosomes, a high-throughput chromatin conformation capture (Hi-C) experiment was conducted, resulting in 248 966 127 read pairs with a total base number of 74.59 Gb. The reads quantification showed that 88.4% of reads had a quality score (Q) of at least 30, and 53.2% of uniquely mapped reads were valid for chromosome interaction analysis (Supplementary Data Tables S2 and S3). Using the heterozygosity and All-HiC software, 99.5% of contigs (14 788) were aligned onto 18 chromosomes, resulting in a total genome size of 1 027 094 429 with the scaffold N50 of 64.4 M (Fig. 1B, Supplementary Data Tables S4–S6). The intragenomic comparison of the 18 chromosome sequences of *S. glauca* showed good intensity and consistency of the two sets of haplotypes (Fig. 1C and D).

### Annotation of *S. glauca* genome and phylogenetic analysis of sequenced species of Chenopodiaceae

The transcriptome of *S. glauca* was sequenced using RNA-seq libraries from 20 different tissues and was mapped to the genome assembly to identify transcription regions along the chromosomes. To supplement the annotation process, the genomes of close relatives such as *C. quinoa*, *B. vulgaris*, and *Solanum lycopersicum* (tomato), as well as other typical species, including **Amborella* trichopoda*, *Oryza sativa* L., *Arabidopsis thaliana*, *Eutrema salsugineum*, *Brassica rapa*, *Brassica oleracea*, *Vitis vinifera* (from Genoscope), and *Populus trichocarpa* were used as references. A total of 54 761 genes were annotated in the *S. glauca* genome (Fig. 2A, Table 1). The genome assembly was evaluated using 1375 BUSCO groups and by searching against the annotated protein sequences, with 91.80% (1262) of the total searched BUSCOs being complete, 27.3% being single-copy (375), 27.30% duplicated, and 3.20% fragmented. Only 69 BUSCOs (5% of the total) were missing (Supplementary Data Table S7), indicating the high quality of the genome release.

**Table 1:** Allele-defined annotation of *S. glauca* genome.

Chr. ID	No. of genes	No. of alleles in haplotype A	No. of alleles in haplotype B	No. of tandem duplicated genes	No. of dispersed duplicated genes
Chr1	3166	2941	2900	47	621
Chr2	3126	2935	2928	41	726
Chr3	3337	3070	3099	57	782
Chr4	3402	3154	3072	33	728
Chr5	3049	2875	2828	46	602
Chr6	3767	2762	3518	34	506
Chr7	2877	2681	2690	74	545
Chr8	2506	2330	2329	20	396
Chr9	1716	1463	1568	27	333
Total	26 946	24 211	24 932	379	5239

The identification of alleles from two sets of haplotype chromosomes was determined by analyzing gene homology and their respective locations on the chromosome.

Using gene homology and chromosome location, we identified the alleles from two haplotypes, resulting in 26 946 gene loci defined along nine pairs of homologous chromosomes, with 24 211 alleles on haplotype A and 24 932 on haplotype B. Among them, 379 were tandem duplicated genes, and 5239 were dispersed duplicated genes (Table 1). An analysis was conducted to study the orthologous gene families among *S. glauca*, *A. thaliana*, *B. vulgaris*, *O. sativa*, and *V. vinifera*. This analysis resulted in the identification of 17 806 clusters and 3737 singletons in *S. glauca* (Fig. 2B, Supplementary Data Table S8). The synteny between two sets of *S. glauca* haploid genomes and the genomes of *A. thaliana*, *B. vulgaris*, *O. sativa*, and *V. vinifera* was also analyzed based on the orthologous layout along the genomes. The results showed that the genome of *S. glauca* exhibited better synteny with the genome of *B. vulgaris*, which has the closest taxonomical relationship with *S. glauca* (Fig. 2C, Supplementary Data Fig. S1).

**Figure 3:**
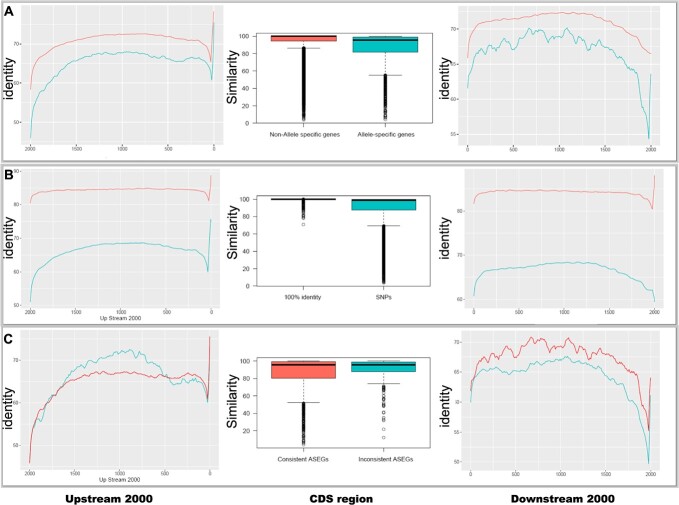
Gene features of allele-specific expression (ASE) genes and non-allele-specific expression (nASE) genes. Sequence identity of upstream 2000 bp (left panel), coding DNA sequences (middle panel), and downsteam 2000 bp (right panel) of nASE genes vs ASE genes (**A**), ASE genes with 100% CDS identity vs ASE genes with SNPs within CDS region (**B**), and consistent ASE genes vs inconsistent ASE genes (**C**).

Repetitive elements in the genome were identified through BLAST searches against the NCBI NR database, resulting in 1 379 264 repeat fragments with a total length of 733 977 099 bp, representing 70.56% of the genome. The total length of known repeats was 607 936 959 bp (947 996 fragments), with retroelements being the most frequently found (46.98%). A higher proportion of LTR retrotransposons were identified than non-LTR retrotransposons (39.58 vs 5.49%), and Ty3/Gypsy was the most representative type in the LTR retrotransposon category (26.59% of the genome). In addition, 377 777 fragments with a total length of 56 332 911 bp (5.42% of the genome) were identified as tandem repeats. We also identified unique repeats not previously reported, with a total length of 12 282 923 bp, accounting for 1.18% of the genome release (Supplementary Data Table S9). Telomeric sequences were also annotated, as shown in Supplementary Data Table S10.

The Amaranthaceae family is of great interest due to its economic and biological importance. Currently, genome data are available for nine species in this family, including three cultivars of *B. vulgaris*, *Beta patula*, *Amaranthus hypochondriacus*, three assemblies of *C. quinoa*, *Chenopodium pallidicaule*, *S. oleracea*, *Kochia scoparia*, and *S. aralocaspica*, with the latter being the focus of this study. To investigate the evolutionary relationships between *S. glauca* and other Amaranthaceae species, RAxML software was used to generate the phylogenetic tree of these species. The results indicated that the Amaranthaceae and Chenopodiaceae families diverged from their common ancestor around 60 million years ago, resulting in two distinct branches (Amaranthaceae and Chenopodiaceae), as per the APG III classification (Fig. 2D).

### Allele-specific expression in *S. glauca*

The high heterozygosity of *S. glauca* was exploited for allele-aware assembly. Transcriptome analysis revealed potential differential expression between alleles (Supplementary Data Fig. S2). A comparison of sequence identity between allelic gene pairs in different functional regions was conducted to understand the homozygosity. The results indicated that transcribed (gene-body) regions had higher sequence identities than non-transcribed regions (upstream and downstream regions), with exons having significantly higher identities than introns (Supplementary Data Fig. S3). Based on gene expression levels in various tissues and treatments, genes were classified as allele-specific expression (ASE) genes (9% of total genes) and non-ASE genes (91% of total genes) (Supplementary Data Fig. S4). The coding DNA sequences (CDS) identity of non-ASE genes was higher than that of ASE genes, and up- and downstream regions of non-ASE genes also had higher identities than those of ASE genes (Fig. 3A). Of the non-ASE genes, 22% had a 100% identical CDS, making it difficult to detect differential expression with RNA-seq. The remaining 69% of allele pairs had SNPs, but the expression differences were not significant. A similar trend was observed when comparing allele pairs with 100% CDS identity and those with SNPs (Fig. 3B). Among ASE genes, consistent ASE genes were those whose expressions were specific to one allele in all samples and treatments. Inconsistent ASE genes had varying allele-specific expression across samples and treatments. Inconsistent ASE genes had comparable CDS identities with consistent ASE genes, but upstream regions of inconsistent ASE genes had significantly higher sequence identities than those of consistent ASE genes. However, downstream regions showed the opposite tendency. Additionally, the identities of upstream 500–1500 bp regions of consistent ASE genes differed significantly (Fig. 3C). These results indicated that the diversity in promoter sequences might contribute to the consistent expression preference between alleles in the *S. glauca* genome.

**Figure 4:**
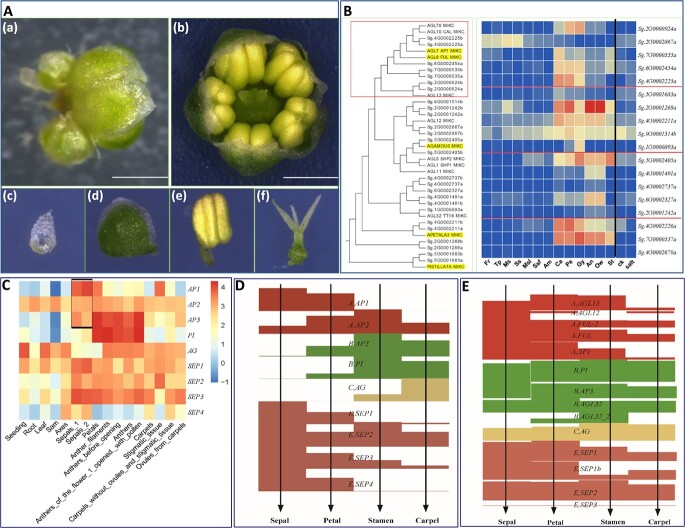
Flower morphology and ABCE genes of *S. glauca*. (**A**) Morphology of *S. glauca* whole flower (a, b), sepal (c), petal (d), stamen (e), and carpel (f). (**B**) Phylogenetic relationship and expressions of *S. glauca* ABCE genes. (**C**) Expressions of ABCE genes in *Arabidopsis*. Expression levels of ABCE genes in *Arabidopsis* were analyzed using normalized read count data obtained from the TraVA database (http://travadb.org/). The heat map represents the log_10_-transformed values of the normalized read counts. (**D**, **E**) Relative expression pattern of ABCE genes in *Arabidopsis* (**D**) and *S. glauca* (**E**).

### Dysfunction of a gene is responsible for the missing petals in *S. glauca*

**Figure 5:**
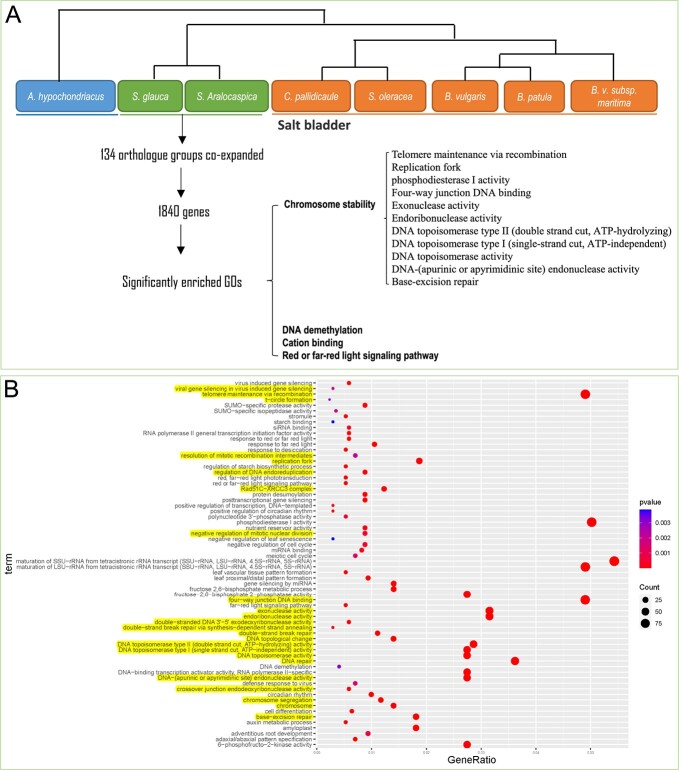
Strategy and results of ortholog expansion analysis of *S. glauca*. (**A**) Significantly expanded orthologs in *S. glauca* were detected compared with other species listed. (**B**) GO enrichment results with the significantly expanded orthologs in *S. glauca*.

**Figure 6:**
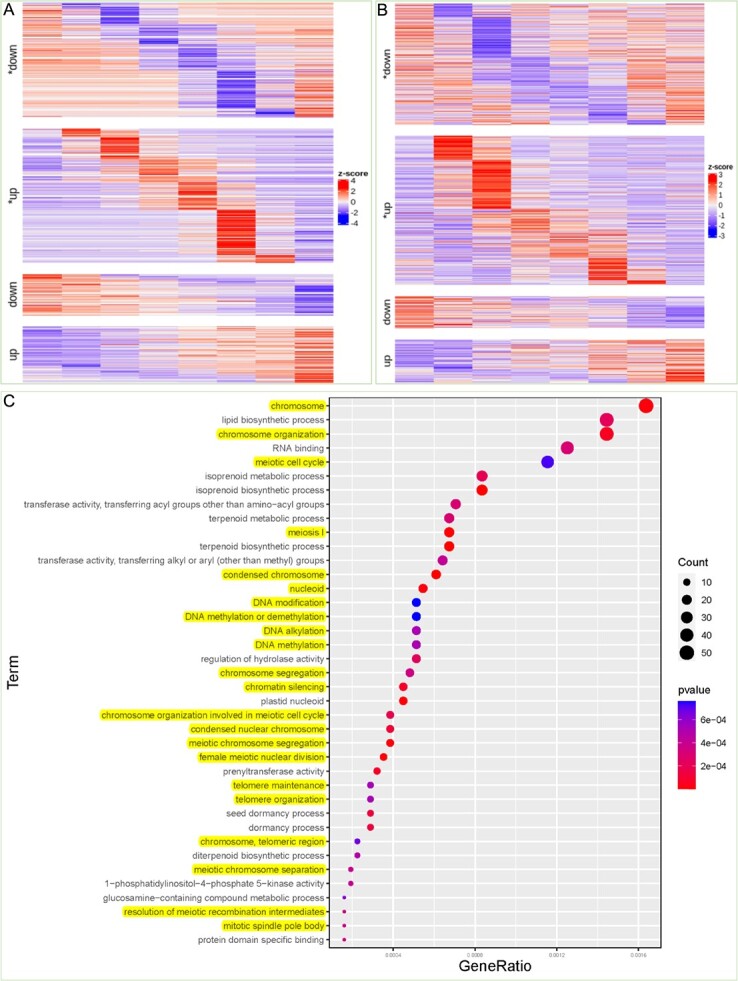
Time-course RNA-seq analysis of *S. glauca* roots and leaves under salt treatment. (**A, B**) Heat maps showing expression of transient down (^*^down), transient up (^*^up), transition down (down), and transition up (up) expression genes in roots (A) and leaves (B) of *S. glauca* under salt treatment. (**C**) Enriched GO terms in transitionally upregulated genes in *S. glauca* leaves.

**Figure 7:**
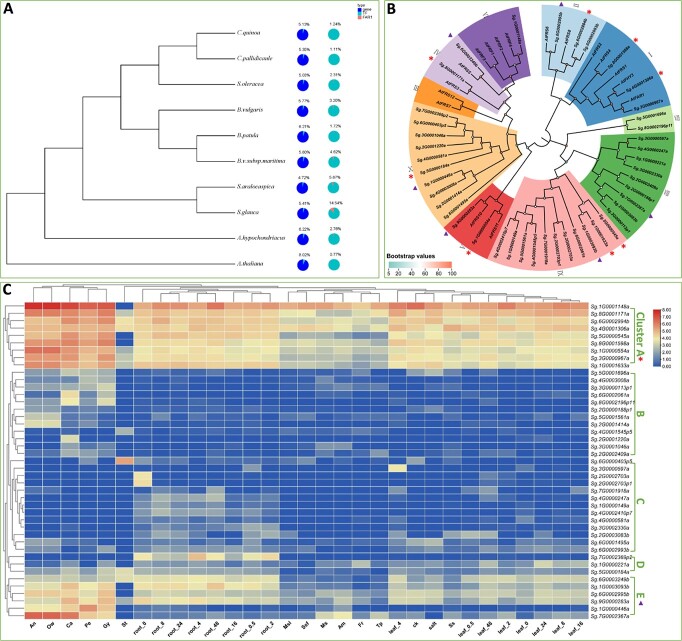
TF analysis and identification of the FAR1 gene family in Amaranthaceae species. (**A**) The chart displays the proportions of TFs in the total genes and *FAR1*s in the total TFs in the *S. glauca* genome. Percentages of TFs and *FAR1*s in various Amaranthaceae species are presented, with *Arabidopsis* used as an outgroup and control. (**B**) Phylogenetic analysis comparing expressed *FAR1* genes from *S. glauca* and *Arabidopsis FAR1* genes. (**C**) Heat map illustrating expression patterns of expressed *FAR1* family genes in *S. glauca*. Expressed *FAR1* genes from *S. glauca* were categorized into groups (I–X) based on the phylogenetic analysis, and further classified into five clusters (A–E) according to their expression patterns. The heat map represents the log_10_-transformed value of FPKM. In the phylogenetic tree figure, cluster A genes are marked with stars and cluster E genes are indicated by triangles.

Flowers have both male (stamen) and female (pistil) reproductive parts, but they typically require cross-pollination from another plant to produce fruits and seeds. Self-fertilization is possible in some species, but it is relatively rare. Flowers evolved to attract pollinators through various visual and olfactory cues, which is why they often have brightly colored petals and distinct fragrances. The typical angiosperm flower consists of four whorls: sepal, petal, stamen, and carpel, although deviations from this arrangement can occur in some species. In *S. glauca*, only three whorls were observed, with the first whorls significantly degraded and the second whorls exhibiting sepal-like characteristics. The third and fourth whorls show the normal phenotype (Fig. 4A). To confirm the sepal-like identity of the second whorl of the *S. glauca* flower, we analyzed the expression of genes involved in photosynthesis and chlorophyll synthesis-related pathways (ath00195, ath00196, ath00710, and ath00860) (Supplementary Data Result 1: Figs 1–4). The clustered heat maps showed that the expression patterns of the genes examined in the second whorls were similar to those of the sepals. The sample clustered trees also showed that the ‘petal’ sample is closely related to photosynthetic tissues, such as leaf samples and stems. In the heat map of ath00860, it is revealed that the expression of *Sg6G0002323a* was high in all green tissues, including ‘sepals’ and ‘petals’. The ortholog of *Sg6G0002323a* in *Arabidopsis* is *AT1G74470*. It encodes a multifunctional protein with geranylgeranyl reductase activity, shown to catalyze the reduction of prenylated geranylgeranyl-chlorophyll a to phytyl-chlorophyll a (chlorophyll a) and free geranylgeranyl pyrophosphate to phytyl pyrophosphate. The protein encoded by *AT1G74470* catalyzes the last step of chlorophyll synthesis (Supplementary Data Result 1: Fig. 5). The expression data from the public database (http://bar.utoronto.ca/eplant/) showed that *AT1G74470* is mainly expressed in photosynthetic tissues, including rosette leaves and stem leaves (Supplementary Data Result 1: Figs 6 and 7). Moreover, *AT1G74470* is expressed primarily in the chloroplast. The high expression of its ortholog *Sg6G0002323a* in sepals indicated that the second whorls of *S. glauca* have obtained sepal identity.

In flower development the ABCE model is widely accepted with the involvement of MADS-box genes. To explore the role of MADS-box genes in flower development in *S. glauca*, we identified 92 MADS-box genes in the *S. glauca* genome (Supplementary Data Figs S5 and S6), including 24 homologs of the ABCE model genes (Fig. 4B, Supplementary Data Table S11). Among these, five A genes (*SgAP1*, *SgFUL*, *SgFUL-2*, *SgAGL12*, and SgAGL13) function for sepals and petals, four B genes (*SgPI*, *SgAP3*, *SgAGL32*, and *SgAGL32-2*) function for petals and stamen, one C gene (*SgAG*) functions for stamen and carpel, and four E genes (*SgSEP1*, *SgSEP1b*, *SgSEP2*, and *SgSEP3*) function in interaction with ABCE proteins. However, the expression pattern of ABCE genes in *S. glauca* showed a non-canonical ABCE expression pattern compared with *Arabidopsis* (Fig. 4C). Specifically, the expression of A genes in *S. glauca* was significantly lower than that of B genes, and only one B gene, *SgPI*, was highly expressed in the second and third whorls, whereas *Arabidopsis* has two B genes (*AtPI* and *AtAP3*) expressed in the second and third whorls. Additionally, the expression of *SgPI* expanded outward to the first whorl (Fig. 4D and E). Thus, the partial dysfunction of A genes in the first and second whorls and the outward expansion of *SgPI* expression may have led to the degradation of sepals in the first whorl and the transformation of petals to sepal-like identity in the second whorl in *S. glauca* flowers.

### The genes responsible for maintaining DNA/chromosome stability showed significant expansion in the *S. glauca* genome

Most *Chenopodium* species are capable of withstanding multiple abiotic stresses, including high levels of salt. However, *Suaeda* species, which are typically found in salt-rich environments such as salt marshes, flats, and coastal regions, have evolved to cope with constant salt pressure. To investigate which gene families were positively selected during evolution in response to this constant salt pressure, an orthologous matrix (Supplementary Data File 1) was created using data from eight *Chenopodium* species. *Spinacia oleracea*, *B. vulgaris*, and *B. patula* were used as control species to identify co-expanded ortholog groups in *S. glauca* and *S. aralocaspica* (Supplementary File 2). The analysis revealed that 134 ortholog groups were significantly expanded in both *S. glauca* and *S. aralocaspica*, encompassing a total of 1840 genes in *S. glauca* (Supplementary Data File 3). Further analysis using Gene Ontology (GO) enrichment identified 66 significant terms related to DNA repair and chromosome stability, DNA demethylation, cation binding, and red/far-red light signaling pathways (Fig. 5, Supplementary Data File 4).

### The genes related to DNA/chromosome stability were upregulated in *S. glauca* leaves in response to salt treatment

To gain insight into the molecular mechanisms underlying salt tolerance in *S. glauca*, we performed RNA-seq analysis on the roots and leaves of 1-month-old seedlings subjected to salt treatment at various time points (0, 0.5, 2, 4, 8, 12, 24, and 48 h). The differentially expressed genes (DEGs) at each time point were subjected to GO enrichment analysis (Supplementary Data Files 8–11). We generated point-plot figures to visualize the enriched GO terms and gain a better understanding of how *S. glauca* roots and leaves cope with salt treatment (Supplementary Data Figs S7 and S8). In the roots of *S. glauca*, we observed significant downregulation of genes related to photosynthesis, photorespiration, and the synthesis and metabolism of secondary metabolites such as terpenoids and flavonoids. Conversely, genes involved in the synthesis of fatty acids, lignin, and cutin were upregulated. The upregulated genes were significantly enriched in GO terms related to the regulation of anthocyanin and syringyl lignin biosynthesis, as well as cutin and lignin biosynthesis processes. Moreover, many response-related GO terms were significantly enriched in the upregulated genes, indicating that *S. glauca* roots mount a complex response to salt stress. GO enrichment analysis also revealed that the jasmonic acid (JA) and abscisic acid (ABA) signaling pathways are involved in the regulation of salt response in *S. glauca* roots. The upregulated genes were significantly enriched in GO terms related to JA and ABA biosynthesis processes (Supplementary Data Fig. S7).

After salt treatment, the photosynthesis-related pathways in *S. glauca* leaves were partially inhibited. The synthesis of wax, very long-chain fatty acids, and sterols was also significantly reduced, while the synthesis of flavonoids, xylan, valine, sphingolipids, terpenoids, proline, lignin, and oxylipins was greatly enhanced. GO terms related to the response to abiotic stress pathways were mainly enriched in upregulated genes, whereas GO terms associated with the cellular response to unfolded proteins, oxidative stress, hypoxia, heat, and amino acid stimulus were significantly enriched in downregulated genes. However, defense response-related GO terms were significantly enriched in both up- and downregulated genes. The JA and ABA pathways were upregulated in *S. glauca* after salt treatment. Moreover, the pathways related to l-ascorbate peroxidase and ion channel activity were significantly upregulated in *S. glauca* under salt treatment (Supplementary Data Fig. S8).

To gain insight into the temporal changes in gene expression, we conducted an impulse analysis on the RNA-seq data. The DEGs were classified into four categories, namely transient up, transition up, transient down, and transition down (Fig. 6A and B). The GO enrichment analysis results for each category of genes in the roots and leaves of *S. glauca* are shown in Supplementary Data Figs S9–16. Notably, the transition up genes were identified as a stable response mechanism for *S. glauca* to cope with salt stress. In the roots, the transition-upregulated genes were mainly enriched in the extracellular matrix, second messenger mediated signaling, autophagy, calcium-mediated signaling, methylated histone, hormone activity binding, and cohesin complex terminology. However, in the leaves, the transition-upregulated genes were mainly associated with DNA repair and chromosome stability, along with GO enrichment related to lipid biosynthetic process and isoprenoid metabolic process. These results are consistent with the previous analysis of the *S. glauca* gene family expansion (Fig. 6C).

### The *FAR* gene family was significantly expanded in *S. glauca*, while the involvement of *FAR1* in salt tolerance needs to be further confirmed

TFs play a critical role in plant responses to biotic and abiotic stresses. In the genome of *S. glauca*, a total of 1761 TFs were identified and classified into 79 families based on the *Arabidopsis* reference genome (Supplementary Data File 5). Comparatively, *Arabidopsis* had 2217 TFs identified using the same identification pipeline [27, 28]. Interestingly, the far-red-impaired response 1 (*FAR1*) transcription family (TF) family was found to be the most abundant in *S. glauca* compared with *Arabidopsis*. Specifically, 257 *FAR*s were identified in *S. glauca*, accounting for 14.3% of the TFs, whereas *Arabidopsis* only had 17 *FAR*s, representing 0.7% of the *TF*s (Supplementary Data File 6). These observations suggest a significant expansion of the *FAR1* gene family in *S. glauca*. To determine whether this expansion is unique to *S. glauca* or occurs more broadly in the *Amaranthaceae* family, TF families of nine sequenced species of Amaranthaceae were examined. The results, presented in Supplementary Data File 7, revealed a significant expansion of *FAR1* TFs in both *S. glauca* and *S. aralocaspica* (Fig. 7A). These findings are consistent with the results of the gene expansion analysis, which identified significant enrichment of genes related to red or far-red signaling pathways in the co-expanded genes of *S. glauca* and *S. aralocaspica*. However, gene expression analysis of the *FAR1* family showed that most of the *FAR1* genes were not expressed in various tissues, including roots and leaves, under normal conditions and salt tolerance. Among the 257 *FAR* genes analyzed, only 44 exhibited expression, which were classified into nine clades based on their phylogenetic relationships (Fig. 7B). Furthermore, these genes were grouped into five clusters according to their expression patterns (Fig. 7C). Notably, two gene clusters, clusters A and E, displayed relatively higher expression levels, primarily observed in floral tissues. Cluster A genes were found to be expressed in floral tissues and leaves and root tissues under both normal and salt-stress conditions, whereas cluster E genes exhibited relatively higher expression exclusively in roots under normal and salt-stress conditions (Fig. 7C). Nevertheless, there is insufficient evidence to suggest that the *FAR1* genes were specifically induced by salt treatment. In summary, our results demonstrate a significant expansion of the *FAR1* gene family in *S. glauca*. However, evidence supporting the involvement of red/far-red signaling pathways in the salt resistance of *Suaeda* species is insufficient, as most *FAR1* genes were not expressed and their expression was not induced by salt tolerance.

## Discussion

Crops play a crucial role in providing us with essential resources such as food, clothing, shelter, and transportation. However, the area of arable land has been decreasing due to various human activities, natural disasters, and climate impacts, leading to a severe impact on crop production [29–31]. Soil salinization is a significant factor that has worsened the food crisis and posed a threat to food security. Since most crops are glycophytes, they cannot be grown in high-salinity land, and improving such lands is expensive. On the other hand, improving crops' salt tolerance through genetic breeding is a cost-effective and efficient approach [29–31]. The primary strategy for enhancing crop salt tolerance is to identify and utilize the genetic resources within crop species. However, many crops lack germplasm resources with high salt tolerance, which limits the extent to which significant salt tolerance improvement can be achieved. Genome analysis is crucial for identifying gene resources, functional gene analysis, and further analysis of the mechanism of specific traits. Through genome analysis of some salt-tolerant species, the mechanism of salt tolerance has been preliminarily clarified. *Thellungiella halophila* was the first model plant used for salt tolerance research [32, 33]. It is known that most plant species in the Amaranthaceae family have high salt tolerance [34]. Thus, genomes of species in this family, including *B. vulgaris*, *B. patula*, *A. hypochondriacus*, *C. quinoa*, *C. pallidicaule*, *S. oleracea*, *K. scoparia*, and *S. aralocaspica*, have been sequenced. For example, the genome study of *C. quinoa* revealed that the specialized epidermal salt gland cells (EBCs) are responsible for its salt tolerance, and the specific expression of ion transporters, H^+^-ATPase, and sugar transporters under salt stress conditions are critical for *C. quinoa* to achieve polarized salt isolation from leaf cells to EBCs [13]. *Suaeda glauca*, another halophytic species in the Amaranthaceae (Protochenopodiaceae) family, can tolerate salinity up to 600 mM NaCl [35–37]. However, *S. glauca* has a unique salt-tolerance mechanism compared with other species in this family, as it absorbs salts and stores them in tissue cells instead of the specialized EBCs and salt rejection mechanism [35–37]. In this study, we *de novo* assembled and annotated a chromosome-scale halophyte genome for *S. glauca*. The results showed that the genome sequence is of high quality and the annotation is extensive, making it an valuable reference for *S. glauca*, a potential model plant of halophyte species.

In the management of salt and water relations, the physiology and biochemistry of halophytes play a crucial role. These plants have adapted to high-salinity environments and developed a range of adaptive mechanisms to maintain salt balance and regulate the water content within and outside their cells [38, 39]. Firstly, halophytes possess specialized ion regulation mechanisms. They can accumulate high concentrations of salts without experiencing toxicity and compartmentalize salts within specific cells or tissues to prevent interference with critical cellular processes. One mechanism utilizes specialized structures such as salt glands and salt bladder cells to excrete excess salts [40]. Secondly, halophytes demonstrate adaptability in water regulation. They often have specialized structures that regulate water evaporation, such as thick leaves, reduced stomatal density to minimize water loss, and unique parenchyma and xylem structures to maintain water stability [41]. Additionally, halophytes are capable of maintaining proper water absorption and transport in the soil to preserve cellular water balance. At the biochemical level, halophytes synthesize a range of antioxidants and protective proteins to counteract oxidative stress and cellular damage caused by high salinity. These compounds help maintain normal metabolic activities and prevent salt-induced harm to cellular structures and functions [39, 40, 42]. *Suaeda glauca* is a halophytic plant species known for its remarkable salt tolerance. It belongs to the genus *Suaeda*, which comprises several halophyte species found in saline environments worldwide. Unlike other well-known halophytes, whose salt tolerances are attributed to salt rejection and secretion, a large number of salt ions are absorbed by the roots and retained in the tissue cells of the *Suaeda* species. However, how do *Suaeda* species survive and develop normally under high salt concentrations? What is the molecular mechanism underlying this salinity tolerance, and how did it evolve? In this study, our genomic and transcriptomic analysis of *S. glauca* provides insight into these questions. First, the expansion analysis of gene families in *S. glauca* revealed that most of the significantly enriched GO terms within the significantly expanded orthologs were related to DNA and chromosome stability (Fig. 5), suggesting that these genes were positively selected after whole-genome duplication events to adapt to their habitat. To confirm whether these genes were selected by salinity selection pressure, we performed an impulse analysis on RNA-seq data, attempting to identify the pathways significantly enriched within the transition-upregulated genes under salt treatment. As expected, this trend was apparent in *S. glauca* leaves, with most of the enriched GO terms related to the maintenance of DNA and chromosome stability (Fig. 6). It is tempting to conclude that the unique salt tolerance mechanism of the *Suaeda* genus compared with other Amaranthaceae plants may be related to the expansion of genes involved in DNA and chromosome stability. To our knowledge, this newly discovered mechanism of salt tolerance in halophytes may provide new implications and direction for improving crop salt tolerance.

The *FHY3*/*FAR1* genes were first identified as components of phytochrome A (phyA)-mediated far-red light signaling in *A. thaliana* [43, 44]. They are the founding members of the FAR1-related sequence (FRS) family of TFs present in most angiosperms [45]. Over the past decade, the gene family has been found to play multiple roles, including in phyA signaling [46], UV-B signaling [47], circadian clock entrainment and flowering [48], chloroplast biogenesis and chlorophyll biosynthesis [49], ABA signaling and responses [50], and branching and plant architecture [51]. A recent study in *Eucalyptus grandis* showed that *FAR1*/*FHY3* may also respond to salt stress, indicating that the gene family has broader functions beyond light signaling [52]. Our study demonstrated that the *FAR1* gene family is significantly expanded in *S. glauca* (Fig. 7), which could be related to the adaptation of this species to its habitat. However, the related GO terms were not significantly enriched in transition-upregulated genes in roots or leaves. Therefore, further research is needed to determine whether the *FHY3*/*FAR1* gene family is involved in salinity tolerance in *S. glauca*.

## Materials and methods

### Plant materials and sequencing

The original *S. glauca* seeds were provided by Yancheng Lvyuan Salt Soil Agricultural Technology Co. Ltd, Yancheng, Jiangsu, Southeast China (http://www.ychpz.com/). After six generations of selection and purification, the *S. glauca* FAFU cultivar was obtained and used for this study. The seeds were germinated in an incubator at 37°C and the seedlings were grown in the greenhouse with a light/dark cycle of 16/8 h. The expression data of *Arabidopsis* ABCE genes were downloaded from the TraVA Database (http://travadb.org/) [53].

### Genome assembly

Genome size estimation for *S. glauca* was carried out using Jellyfish with the *k*-mer pipeline in the JCVI package [54]. The *S. glauca* genome was assembled using a combination of Illumina short-read sequences, PacBio CLR sequences, and high-throughput chromatin conformation capture (Hi-C) technologies, as previously described [55]. The PacBio CLR reads were first corrected and trimmed, and then assembled into initial contigs using the Canu assembler [56] with the following optimized parameters: batOptions = −dg 3 -db 3 -dr 1 -ca 500 -cp 50. To improve the accuracy of the assembly and mitigate random base errors in the PacBio sequencing system, the Illumina short reads were mapped to the initial genome assembly using BWA-MEM [57] with default parameters, and the alignments were polished using the Pilon program [58] with default parameters. A monoploid genome was first assembled based on the Canu initial haplotigs, and redundant contigs were subsequently removed using Khaper (https://github.com/lardo/khaper), a haplotype caller based on the *k*-mer counting algorithm. Misassemblies were corrected using Hi-C signals with the ALLHiC corrector [59], after quality controls and validation of Hi-C raw data were performed using the HiC-Pro pipeline. Next, high-quality Hi-C reads were mapped to the corrected haplotigs, and the Hi-C signals were used to partition haplotigs and determine their orientation using the ALLHiC scaffolding model. Two haplotype-resolved genomes were also assembled using the Canu initial contigs, and misassemblies were corrected using the same approach as the monoploid assembly. The corrected contigs were phased and scaffolded using the ALLHiC polyploid pipeline with the monoploid assembly as a reference to identify allelic contigs. After manual adjustment with Juicebox (https://github.com/aidenlab/Juicebox), the haplotype genomes were anchored onto chromosome-scale assemblies. The genome assembly completeness and synteny between the two haplotypes were assessed using BUSCO [60] and JCVI. Both the raw data and the genome assembly have been deposited at the China National Center for Bioinformation (https://www.cncb.ac.cn/) under the accession numbers subCRA017428 and WGS038631, respectively.

### Genome annotation

The annotation of the *S. glauca* genome assembly followed the pipeline previously developed in a sugarcane project [61]. This involved classifying repetitive sequences using RepeatMasker [62], further classifying some unknown transposable elements using TEclass [63], and identifying tandem repeats using Tandem Repeats Finder (TRF) [64]. LTRs were predicted and clustered using LTR_retriever [65]. For gene annotation, we used the homology-based and *de novo* methods in the MAKER pipeline [65]. RNA-seq reads were assembled by Trinity [66] with *de novo* assembly and genome-guided assembly. The first round of the MAKER pipeline was used to predict the genes, and the homologs of close relative species were used in the second round of MAKER to improve the annotation quality.

### Phylogenetic tree construction

The genome sets were obtained from NCBI and Phytozome (https://phytozome-next.jgi.doe.gov/), and OrthoFinder [67] was used to identify orthogroups and single-copy ortholog sequences with default parameters. MUSCLE [68] was used for multiple sequence alignment to identify conserved single-copy orthologs, which formed the basis for accurate phylogenetic reconstruction and divergence time estimation. After selecting the appropriate model with ProTest [68], RAxML [69] was used to construct the phylogenetic tree. The MCMCtree [70] program, which employs a Markov chain Monte Carlo algorithm, was used to estimate species divergence times. Gene family expansion and contraction across the specified phylogenetic tree were estimated using CAFE [71], with the global parameter lambda estimated by maximum likelihood. Finally, the phylogenetic tree was visualized using EVOLVIEW (https://www.evolgenius.info/evolview/).

## Acknowledgements

We thank Chunyin Zhang for providing the original seeds of *S. glauca*. We also thank the editors and reviewers for their valuable feedback on this manuscript. This work was supported by the National Natural Science Foundation of China (32170380), the Science and Technology Innovation Project of Pingtan Institute of Science and Technology (PT2021001), and the Postdoctoral Foundation of China (2018 M642550).

## Author contributions

Y.C. and Y.Q. conceived and designed the research. J.S. and M.J. conducted genome assembly and annotation. J.S., Y.L., W.L., and P.Z. conducted other bioinformatic work. B.H., C.D., K.Y., Z.Li., F.D., L.W., and L.C. performed laboratory experiments. H.W., M.A., S.W., S.C., C.P., and Y.Q. contributed to critical discussions on the work. Z.Luo. wrote a draft of the introduction, M.J. wrote the materials and methods section, and Y.C. wrote the other sections and was responsible for writing. M.A. and Y.Q. revised the manuscript. All authors discussed the results, contributed to manuscript preparation, and approved the final version of the manuscript.

## Data availability

All relevant data are contained within the article or supplementary materials.

## Conflict of interest

The authors declare no conflict of interest.

## Supplementary data

Supplementary data is available at *Horticulture Research* online.

## Supplementary Material

Web_Material_uhad161
