# Supplementary material for: Chromosome-scale genome sequence of Suaeda glauca sheds light on salt stress tolerance in halophytes
Source: Hortic Res. 2023 Aug 10;10(9):uhad161. doi: 10.1093/hr/uhad161 (PMC10506132; doi:10.1093/hr/uhad161)
Supplement: Web_Material_uhad161 [file web_material_uhad161.zip › 3.Supplementary Figures-16 Figures_revised_20230530.docx]

Supplementary figures


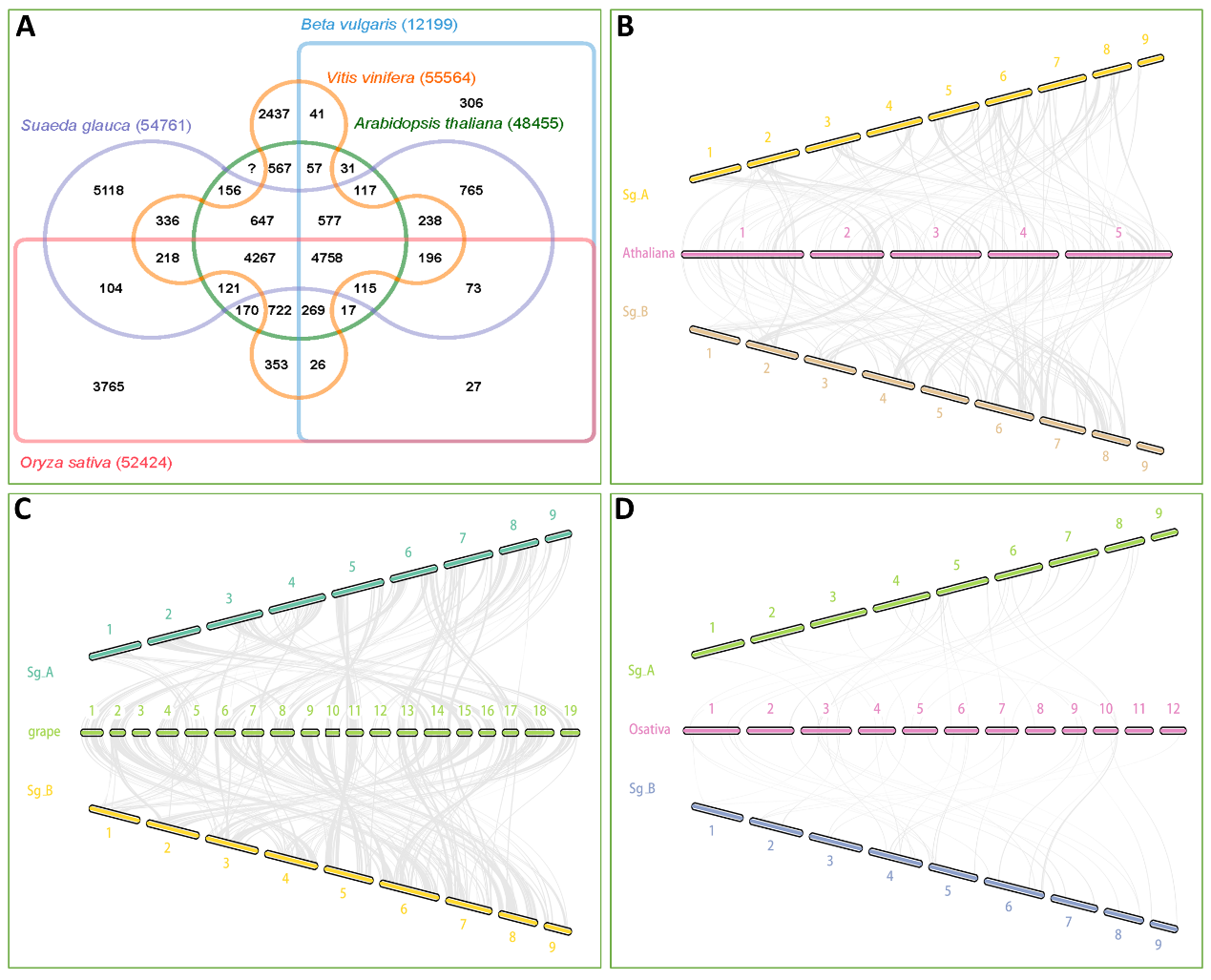


**Figure S1. Orthologous and synteny analysis of the *S. glauca* genome compared to the genomes of *A. thaliana*, *B. vulgaris*, *O. sativa*, and *V. vinifera*.** (A) Venn diagram depicting orthologous gene families shared among *S. glauca*, *A. thaliana*, *B. vulgaris*, *O. sativa*, and *V. vinifera*. (**B, C, D**) Synteny analysis of two sets of haploid genomes from *S. glauca* (Sg) compared to the genomes of *A. thaliana* (B), *V. vinifera* (C), and *O. sativa* (D).


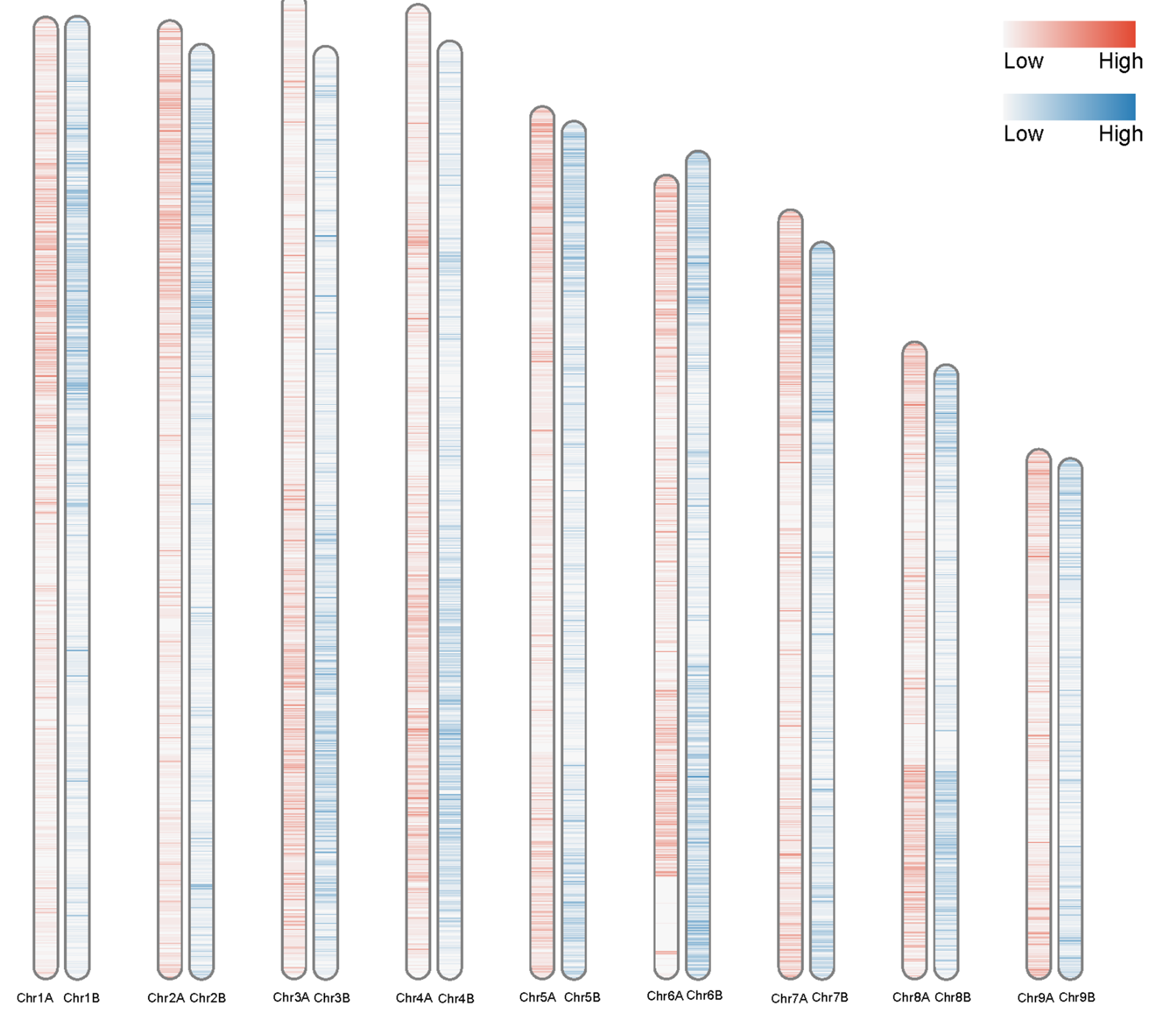


**Figure S2. Differential expression of alleles in *S. glauca.*** The heatmap displays gene locus expression levels along the chromosomes.


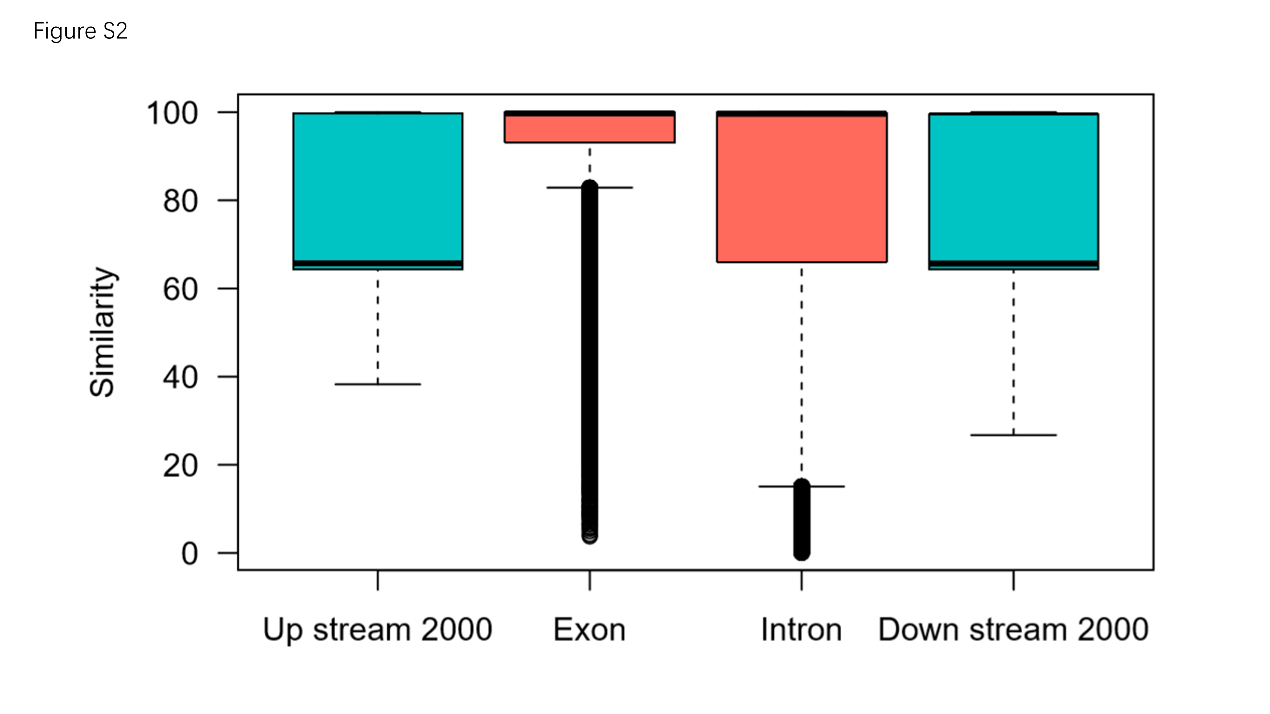


**Figure S3. Allele sequence identity in *S. glauca*.** The DNA identities of upstream 2000 bp, exon, intron, and downstream 2000 bp are shown.


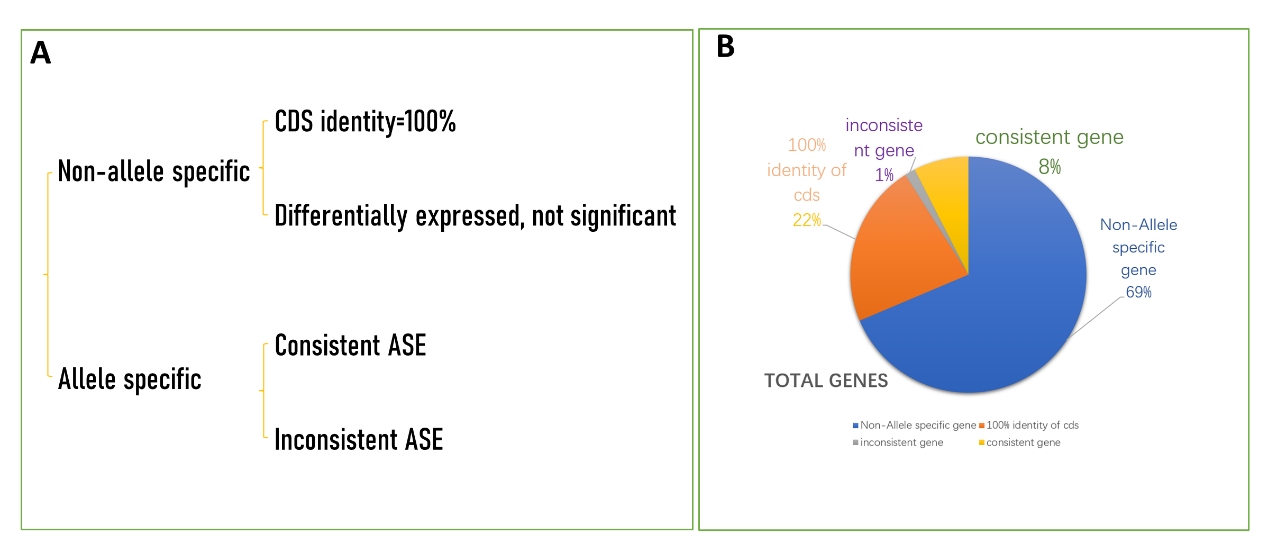


**Figure S4. Allele-specific expression analysis in *S. glauca*.** (A) Non-allele-specific expression (nASE) and allele-specific expression (ASE) gene categories. (B) Pie chart displaying the gene categories in the ASE analysis. The numbers indicate the percentage of different gene categories in the *S. glauca* genome.


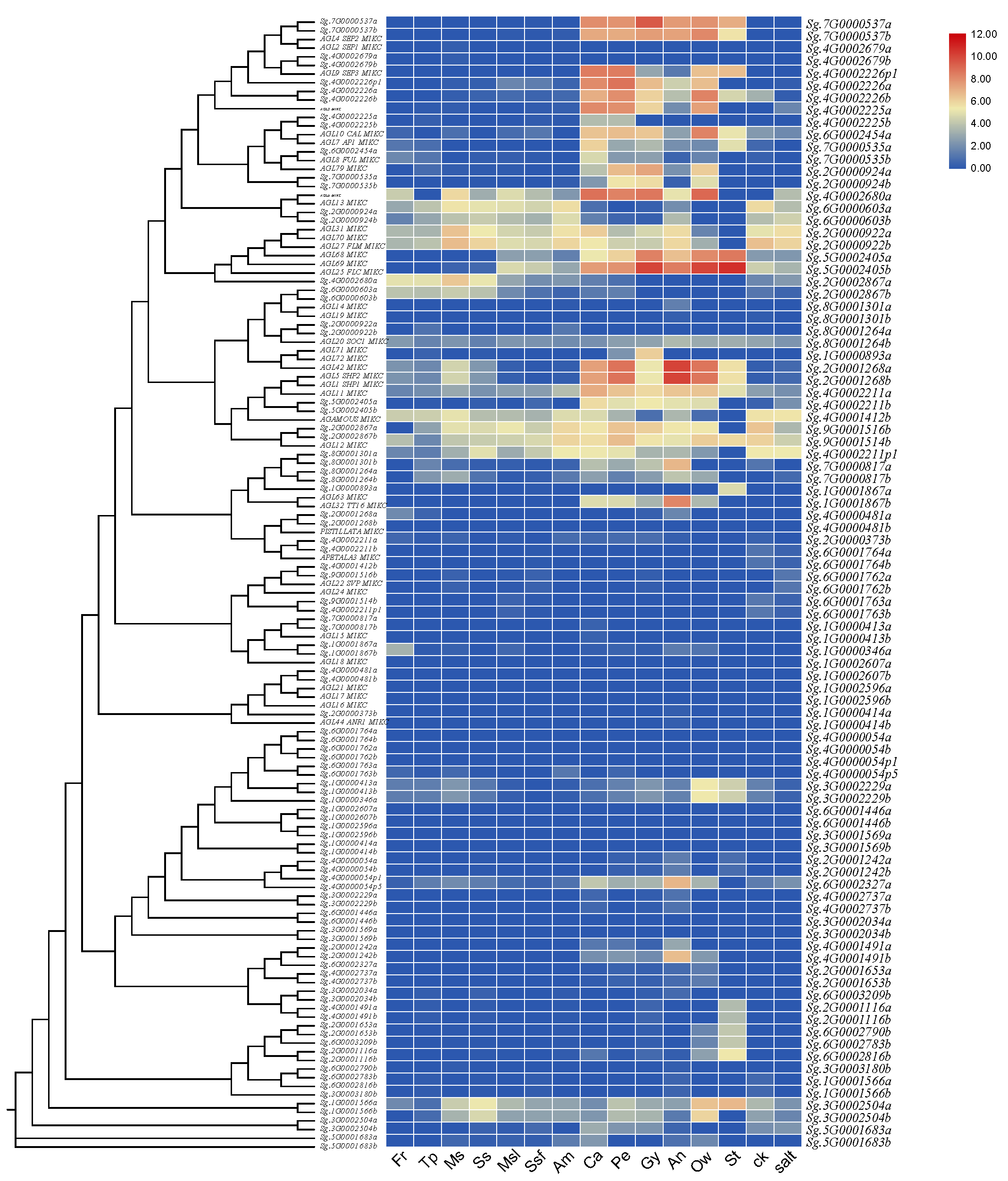


**Figure S5. Identification and Expression Analysis of MADS-box Genes in the *S. glauca* Genome.** The figure displays the identification and expression patterns of MADS-box genes in the *S. glauca* genome. A phylogenetic tree was constructed using the coding sequence (CDS) sequences of the MADS-box genes. The heatmap visualizes the expression levels of the MADS-box genes, represented by log2-transformed values of FPKM.


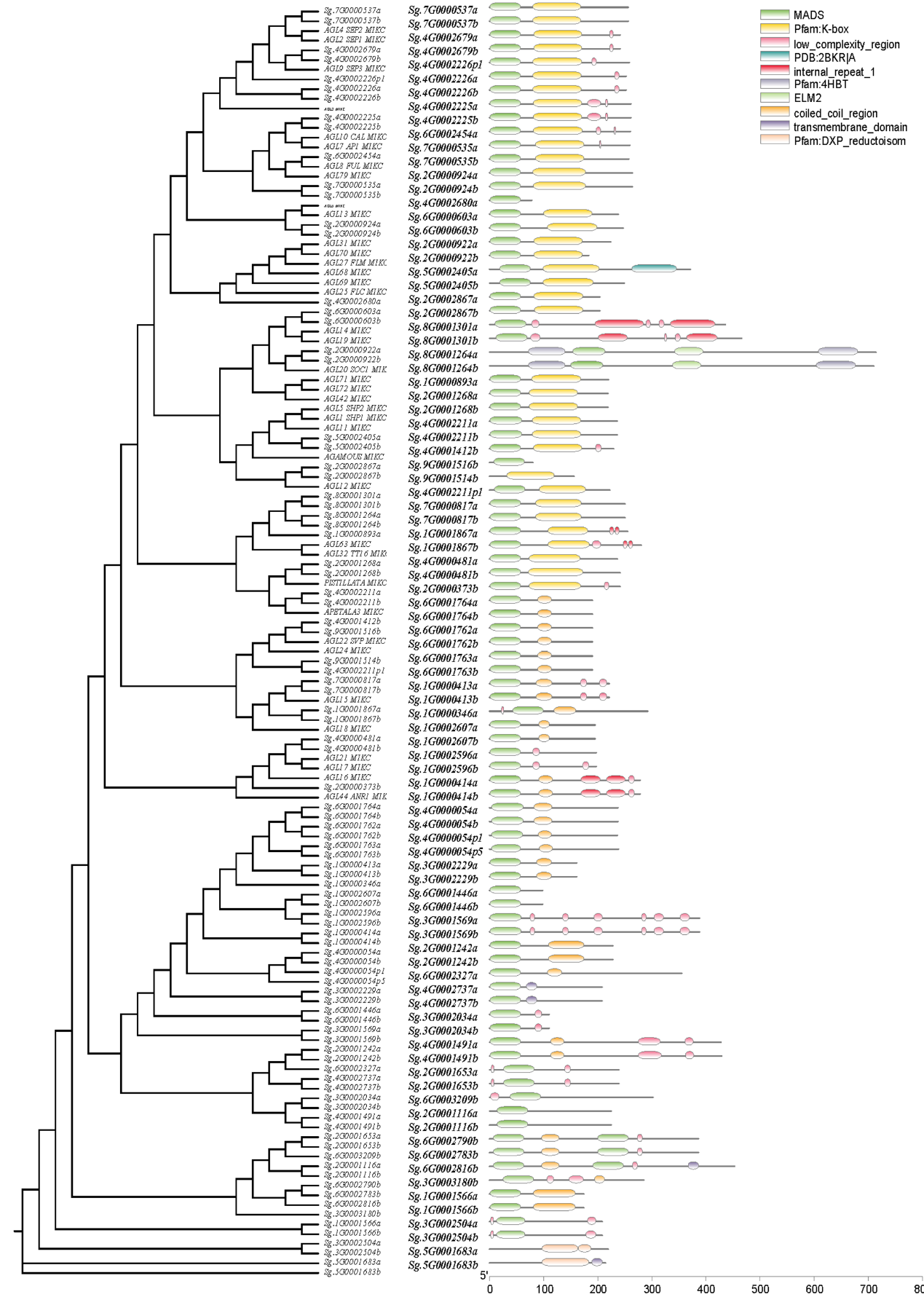


**Figure S6. Conserved domains of FAR1 family genes of *S. glauca*.** The same phylogenetic tree as Figure S5 was shown on the left again to indicate phylogenetic relations ship between the FAR1 genes and the motifs of the *S. glauca* FAR1 genes were depicted on the left.


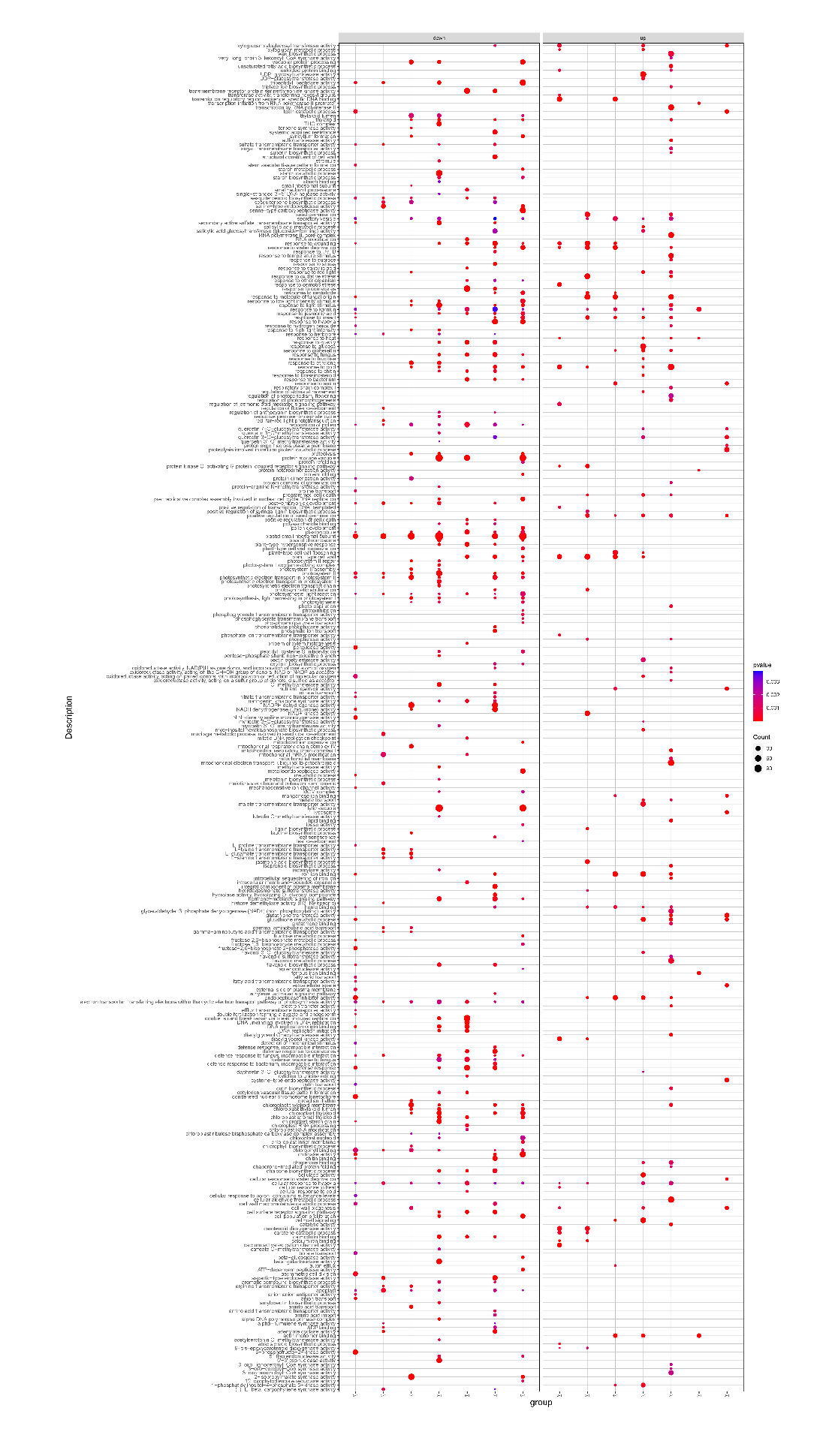


**Figure S7. Time-course GO enrichment analysis with differentially expressed genes in roots under salt treatment.**


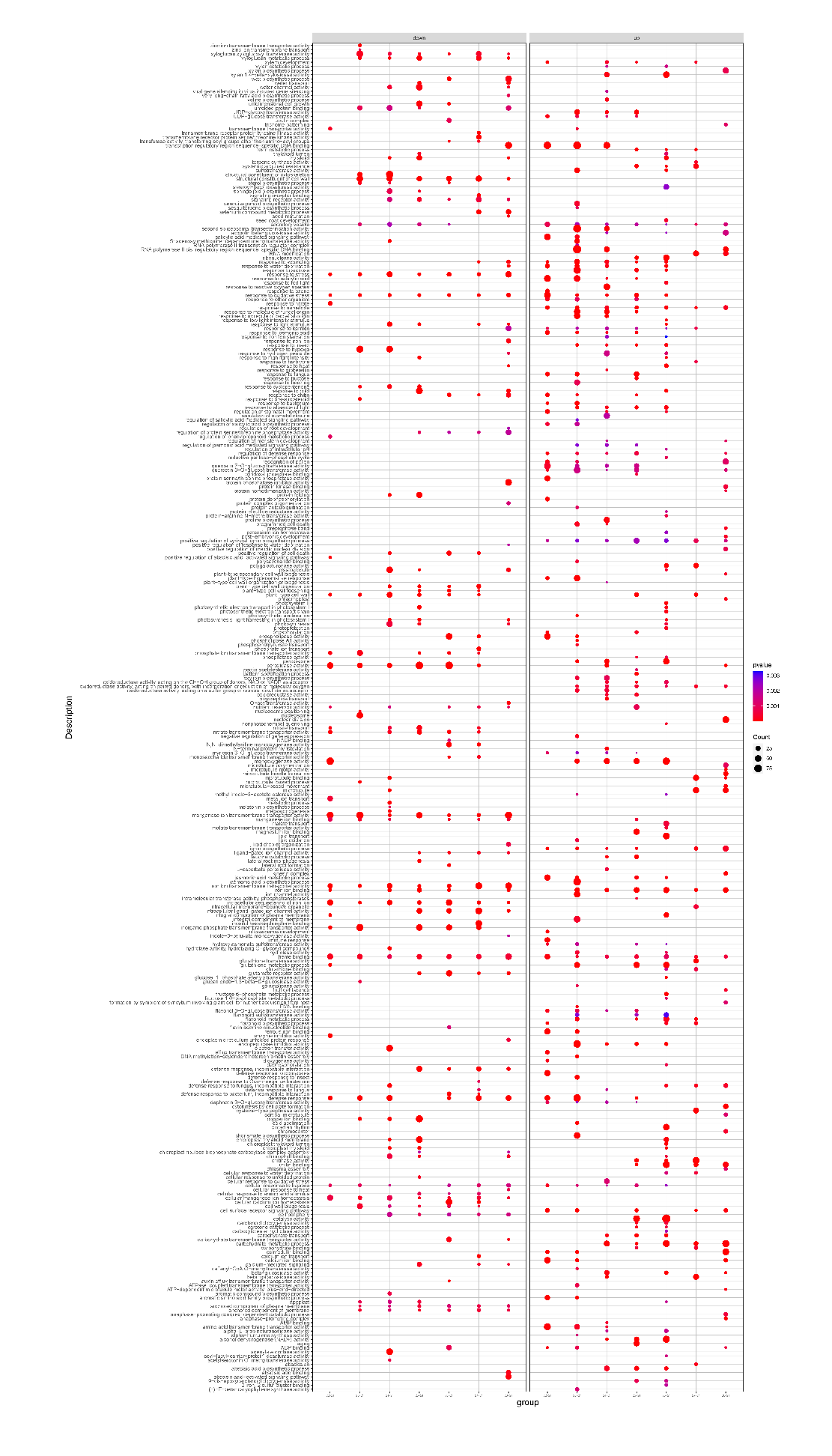


**Figure S8. Time-course GO enrichment analysis with differentially expressed genes in leaves under salt treatment.**


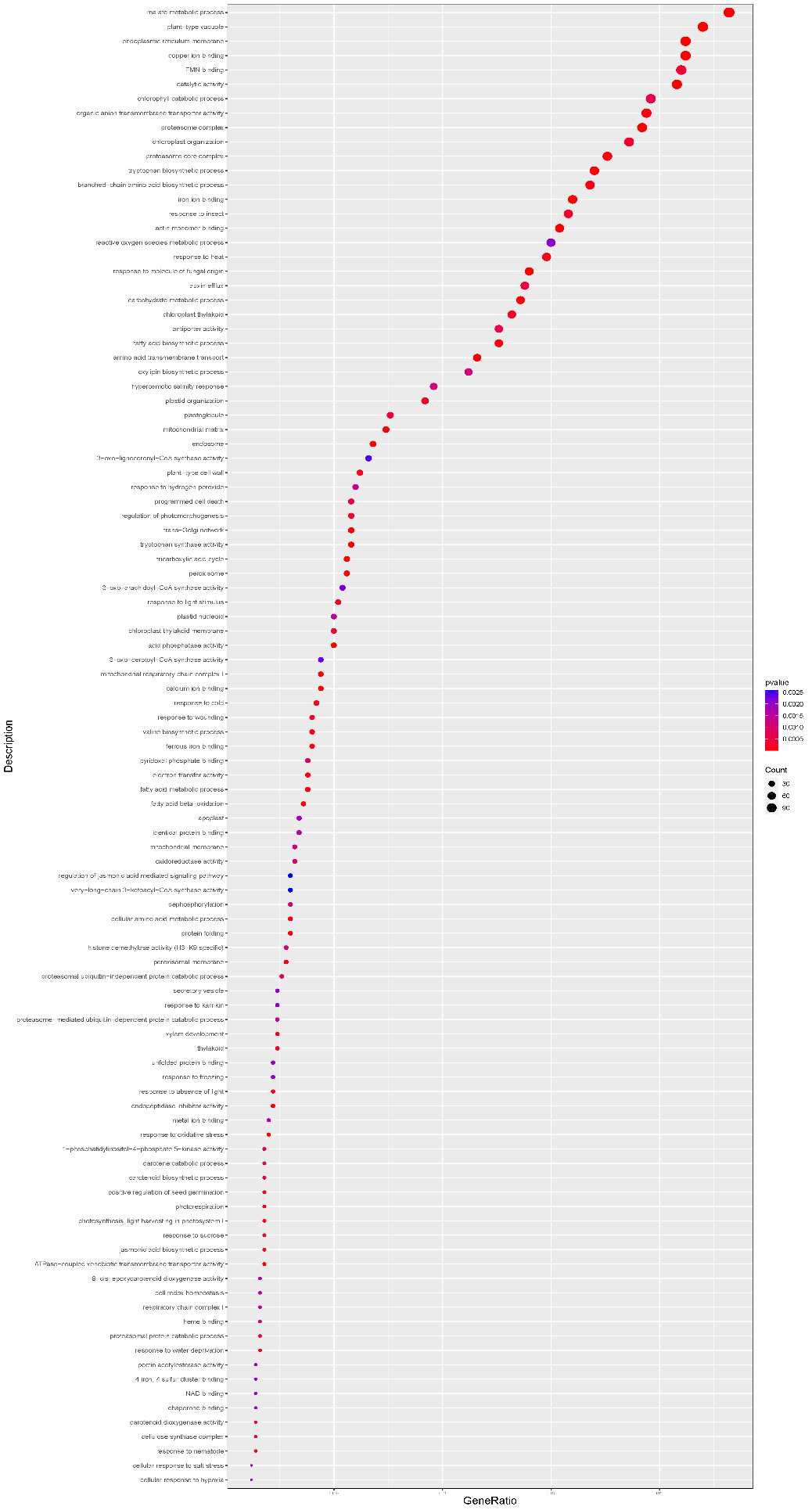


**Figure S9. Enriched GOs with transiently up-regulated genes in roots under time-course salt treatment.**


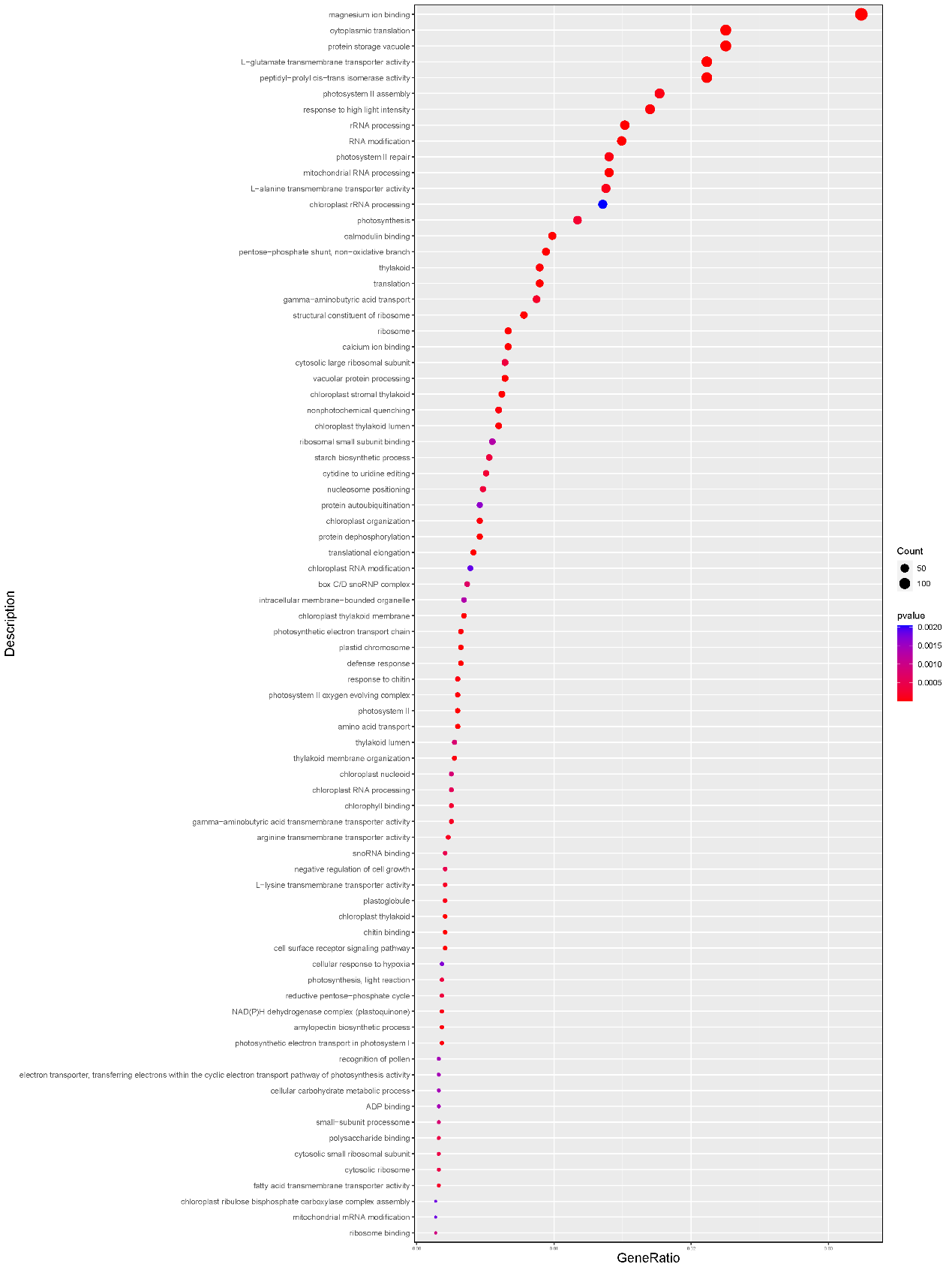


**Figure S10. Enriched GOs with transiently down-regulated genes in roots under time-course salt treatment.**


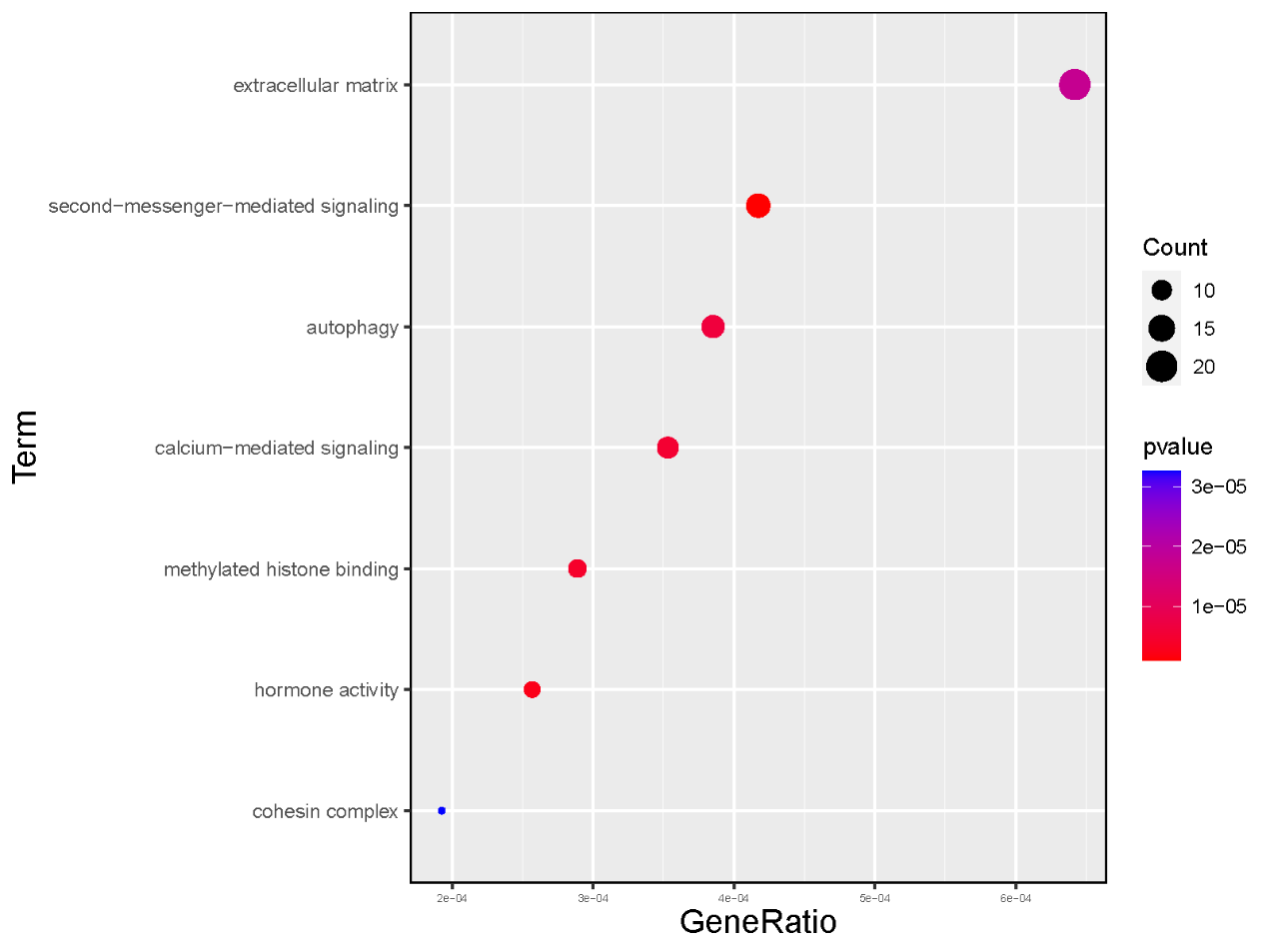


**Figure S11. Enriched GOs with transitionally up-regulated genes in roots under time-course salt treatment.**


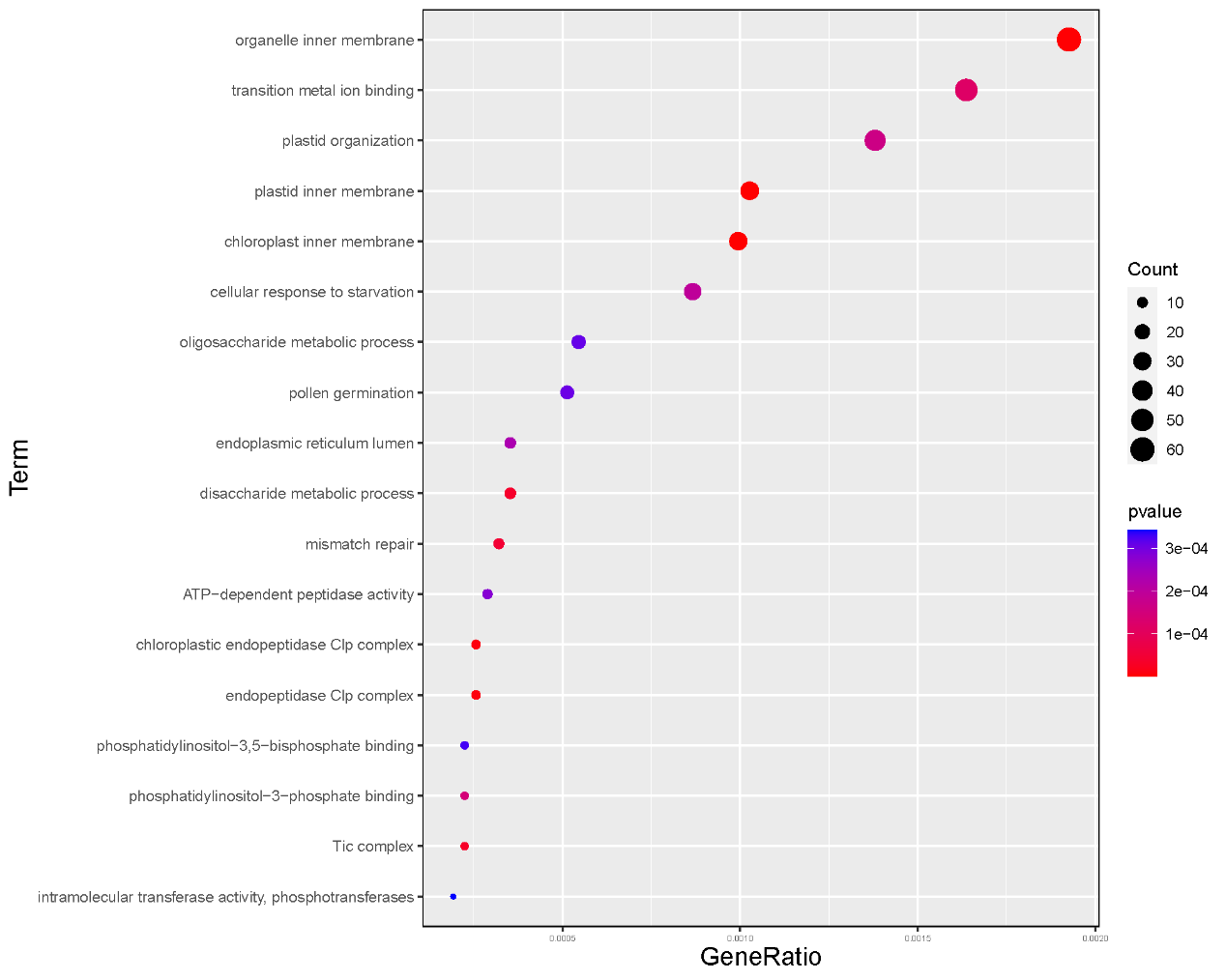


**Figure S12. Enriched GOs with transitionally down-regulated genes in roots under time-course salt treatment.**


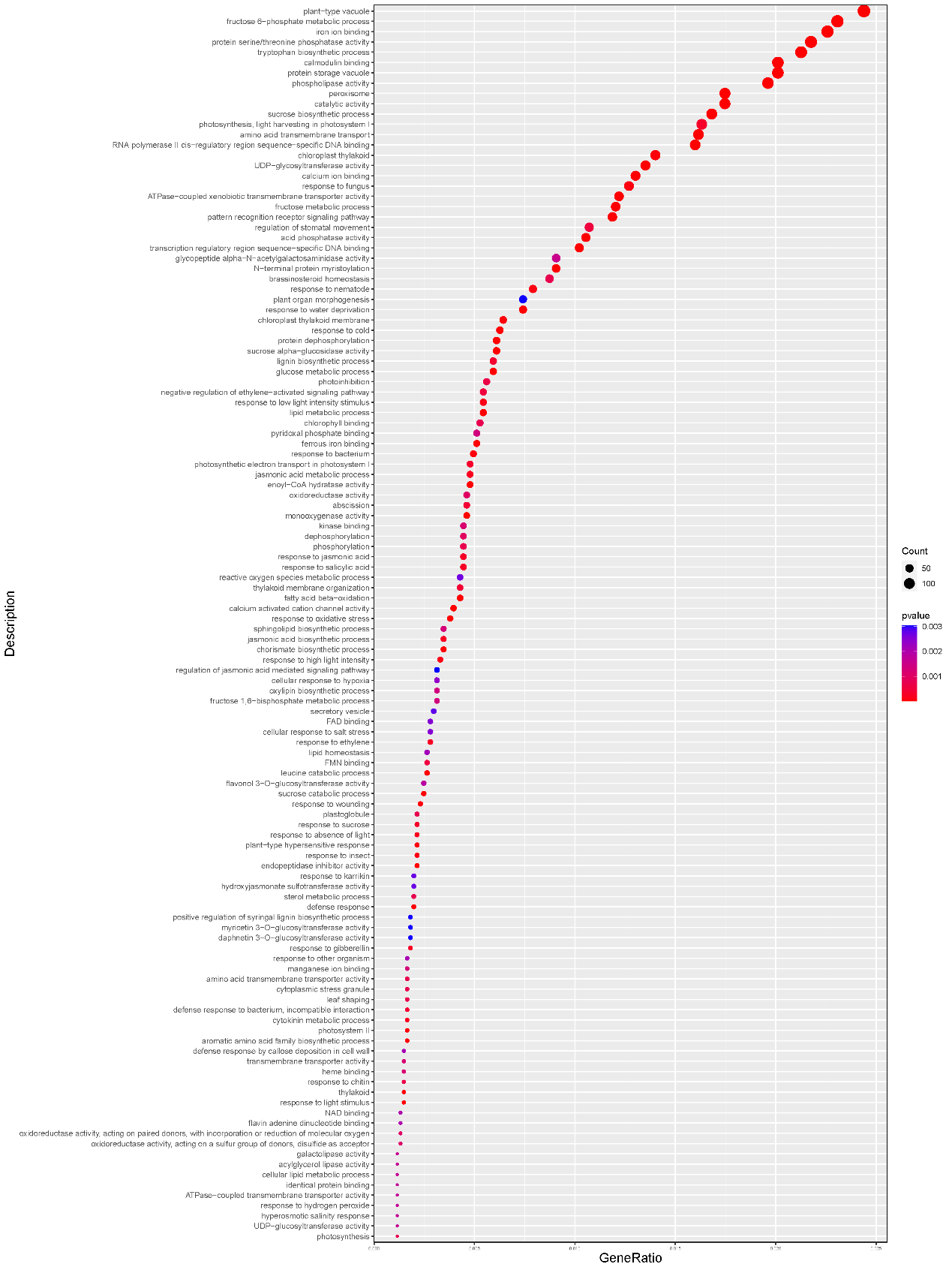


**Figure S13. Enriched GOs with transiently up-regulated genes in leaves under time-course salt treatment.**


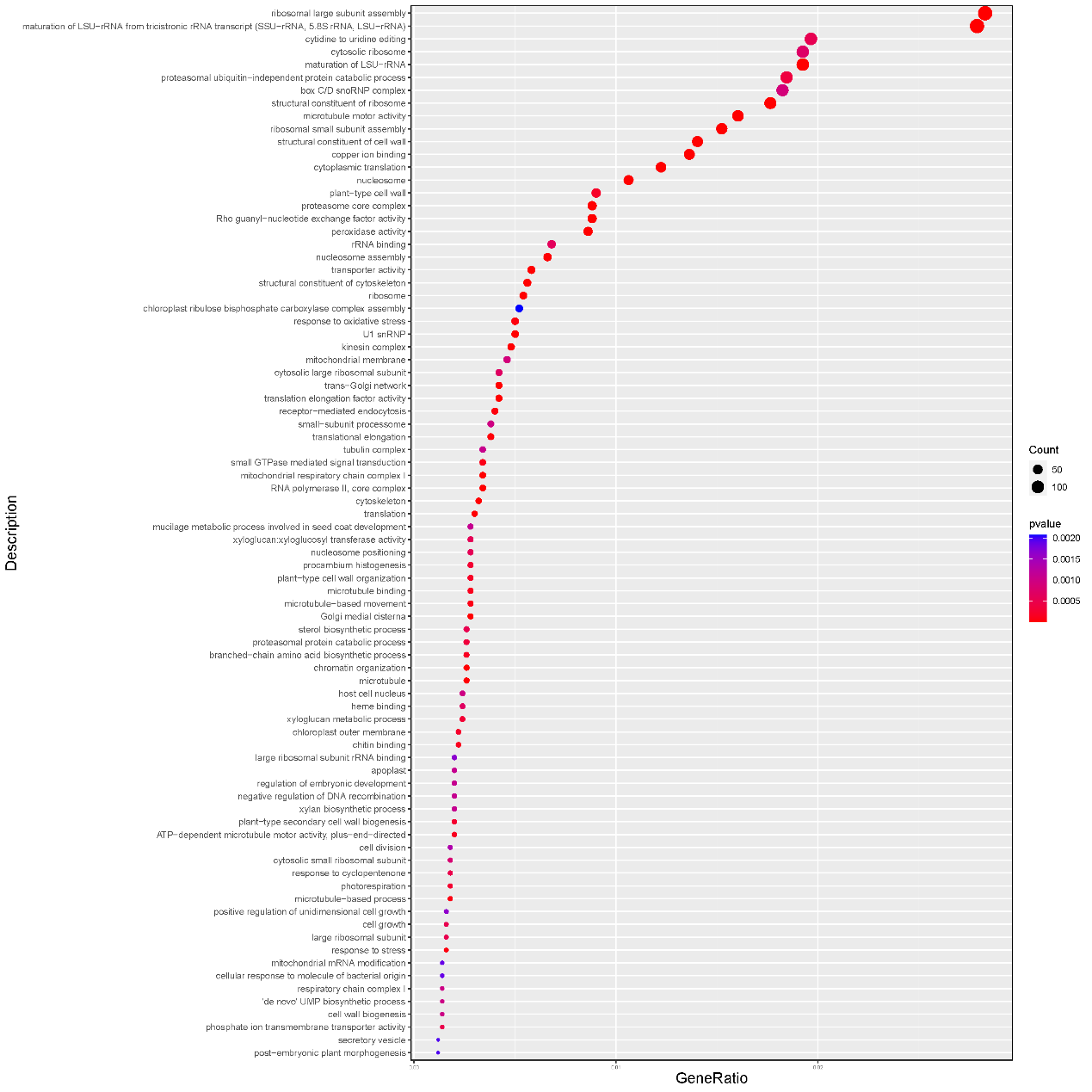


**Figure S14. Enriched GOs with transiently down-regulated genes in leaves under time-course salt treatment.**


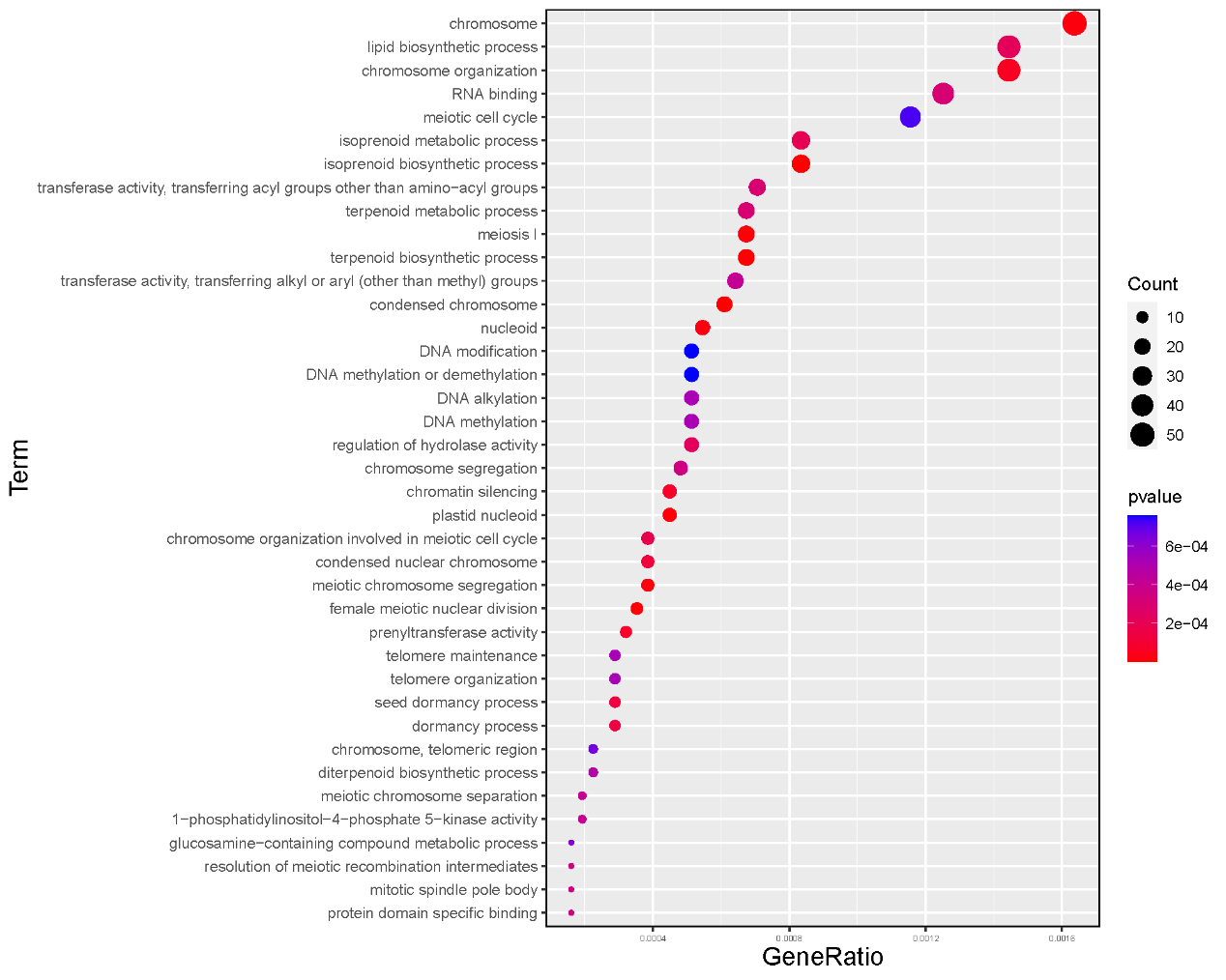


**Figure S15. Enriched GOs with transitionally up-regulated genes in leaves under time-course salt treatment.**


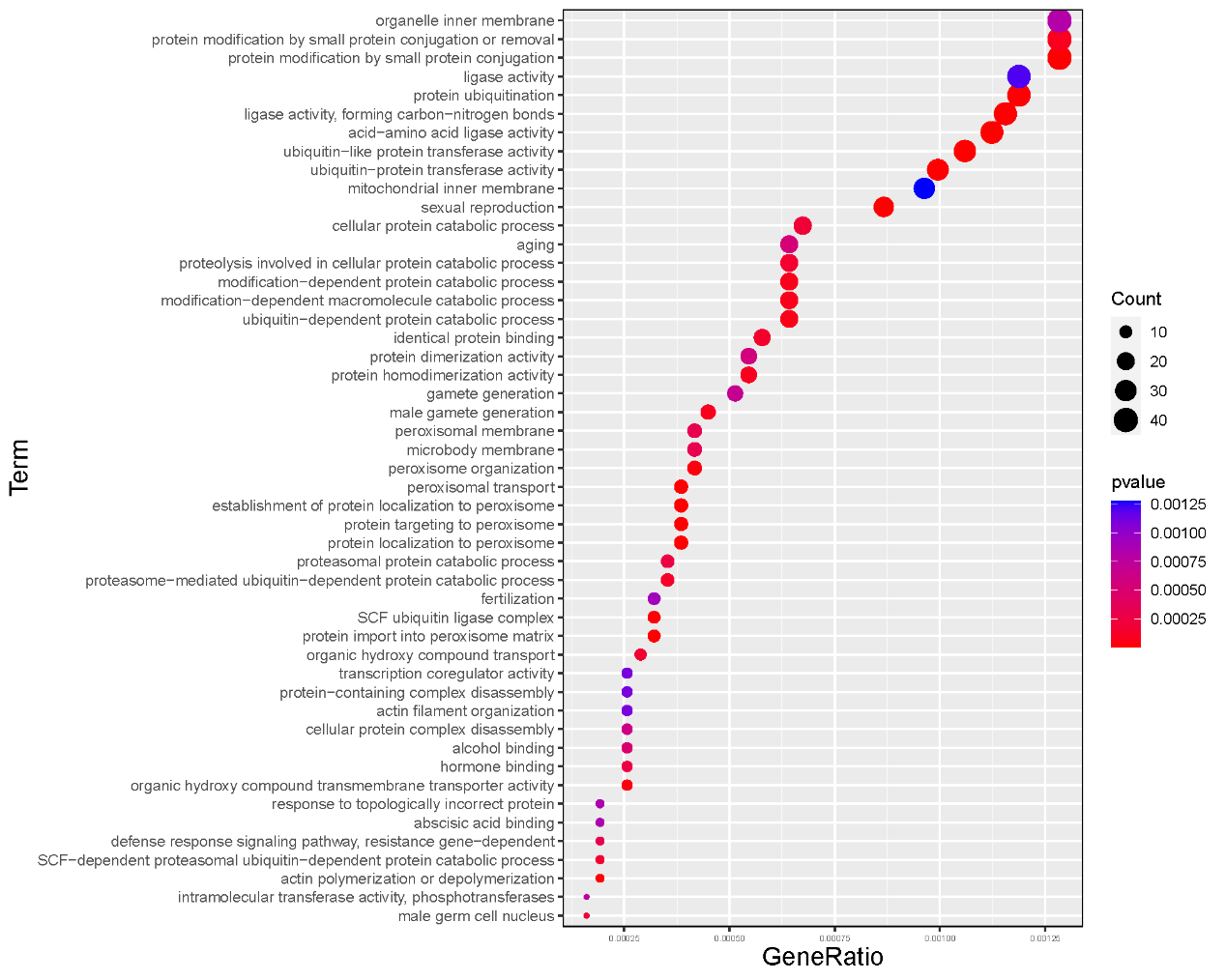


**Figure S16. Enriched GOs with transitionally down-regulated genes in leaves under time-course salt treatment.**
