# Supplementary material for: Chromosome-scale genome sequence of Suaeda glauca sheds light on salt stress tolerance in halophytes
Source: Hortic Res. 2023 Aug 10;10(9):uhad161. doi: 10.1093/hr/uhad161 (PMC10506132; doi:10.1093/hr/uhad161)
Supplement: Web_Material_uhad161 [file web_material_uhad161.zip › 4.Suplementary Tables-11 Tables-Revised_20230530.docx]

**Table S1. The statistics of assembled contigs.**

| **Number of seq** | **15340** |
| --- | --- |
| Min length | 1933 |
| Max length | 7626703 |
| Total Size | 687275827 |
| N90 | 131422 |
| N80 | 221895 |
| N70 | 330416 |
| N60 | 453655 |
| N50 | 600609 |
| Average length | 2373319 |
| Total number (>500bp) | 2896 |
| Total number (>2Kb) | 2895 |

**Table S2. Statistics of Hi-C reads.**

| **Sample** | **BMK-ID** | **Read Pairs Number** | **Base Number** | **%≥Q30** |
| --- | --- | --- | --- | --- |
| Suaeda glauca | L4702-H01 | 248,966,127 | 74,593,690,324 | 88.45 |

**Table S3. Validation of Hi-C reads.**

| Type | Number | Ratio(%) |
| --- | --- | --- |
| Unique Paired Alignments | 7,475,704 | 100 |
| Valid Interaction Pairs | 3,977,440 | 53.2 |
| Dangling End Pairs | 994,670 | 13.31 |
| Re-ligation Pairs | 352,733 | 4.72 |
| Self-cycle Pairs | 759,519 | 10.16 |
| Dumped Pairs | 1,391,342 | 18.61 |

Validation of the Hi-C reads was performed by mapping them to the sample contigs. Only unique paired alignments were considered for data validation, and the analysis revealed that 53.2% of these alignments were valid.

| **Table S4. Statistics of anchored contigs.** |  |
| --- | --- |
| Total number of contigs | 15340 |
| Total length of contigs (bp) | 1027094429 |
| Total number of anchored contigs | 14788 |
| Total length of chromosome level assembly (bp) | 1023646060 |
| Number of unanchored contigs | 552 |
| Length of unanchored contigs | 4925369 |
| Anchor rate (%) | 99.52 |

**Table S5. Anchored contigs and their lengths for the chromosomes.**

| **Chr ID** | **Anchored_ctg** | **Length** |
| --- | --- | --- |
| Chr3A | 912 | 68088806 |
| Chr4A | 976 | 67294906 |
| Chr1B | 975 | 66515229 |
| Chr1A | 844 | 66429904 |
| Chr2A | 888 | 66183624 |
| Chr4B | 1140 | 64809742 |
| Chr2B | 1018 | 64593328 |
| Chr3B | 1041 | 64443754 |
| Chr5A | 888 | 60245902 |
| Chr5B | 896 | 59265822 |
| Chr6B | 726 | 57202922 |
| Chr6A | 690 | 55516602 |
| Chr7A | 734 | 53124425 |
| Chr7B | 723 | 50918097 |
| Chr8A | 573 | 43998169 |
| Chr8B | 628 | 42451120 |
| Chr9A | 535 | 36585467 |
| Chr9B | 601 | 35978241 |

**Table S6. The statistics of anchored scaffolds.**

| Term | HapA Contig | HapB Contig | Total Contig | Total Scaffold |
| --- | --- | --- | --- | --- |
| Number of seq | 7,283 | 8,057 | 15,340 | 570 |
| Min length | 26 | 202 | 26 | 26 |
| Max length | 1,991,044 | 2,989,132 | 2,989,132 | 68,088,806 |
| Total size | 518,672,860 | 508,421,569 | 1,027,094,429 | 1,028,571,429 |
| N90 | 25,000 | 25,000 | 25,000 | 42,451,120 |
| N80 | 59,993 | 50,000 | 53,000 | 50,918,097 |
| N70 | 110,640 | 85,320 | 98,487 | 55,516,602 |
| N60 | 180,121 | 133,825 | 153,610 | 59,265,822 |
| N50 | 272,000 | 195,186 | 229,070 | 64,443,754 |
| Average length | 71,216 | 63,103 | 66,955 | 1,804,511 |
| Total number(>500bp) | 7,282 | 8,056 | 15,338 | 568 |
| Total number(>2kb) | 7,040 | 7,812 | 14,852 | 370 |

**Table S7. Genome integrity evaluation by BUSCO.**

| Complete BUSCOs (C) | 1262 | 91.80% |
| --- | --- | --- |
| Complete and single-copy BUSCOs (S) | 375 | 27.30% |
| Complete and duplicated BUSCOs (D) | 887 | 64.50% |
| Fragmented BUSCOs (F) | 44 | 3.20% |
| Missing BUSCOs (M) | 69 | 5.00% |
| Total BUSCO groups searched | 1375 |  |

Note: The benchmarking of file Sg.anno_v20191205.protein.fasta was summarized using BUSCO notation. BUSCO was run in protein mode.

**Table S8. Orthologous gene families among *Suaeda glauca*, *Arabidopsis thaliana*, *Beta vulgaris*, *Oryza sativa*, and *Vitis vinifera*.**

| Species | Proteins | Clusters | Singletons |
| --- | --- | --- | --- |
| *Arabidopsis thaliana* | 48455 | 15462 | 4566 |
| *Beta vulgaris* | 12199 | 7613 | 2578 |
| *Oryza sativa* | 52424 | 15201 | 13450 |
| *Suaeda glauca* | 54761 | 17806 | 3737 |
| *Vitis vinifera* | 55564 | 15709 | 9437 |

**Table S9. Statistics of repetitive elements in the *S. glauca* genome.**

|  |  |  | **Number** | **Length(Mb)** | **%of repeats** | **%of genome** |
| --- | --- | --- | --- | --- | --- | --- |
| **Total repeat fraction** | |  | 1379264 | 733977099 | 100 | 70.56 |
| **Class I: Retroelement** | |  | 593668 | 488678290 | 66.58 | 46.98 |
|  | **LTR Retrotransposon** | | 349920 | 411781966 | 56.1 | 39.58 |
|  |  | Ty1/Copia | 37686 | 32620215 | 4.44 | 3.14 |
|  |  | Ty3/Gypsy | 185089 | 276652974 | 37.69 | 26.59 |
|  |  | Other | 127145 | 102508777 | 13.97 | 9.85 |
|  | **non-LTR Retrotransposon** | | 157454 | 57154851 | 7.79 | 5.49 |
|  |  | LINE | 126147 | 53643673 | 7.31 | 5.16 |
|  |  | SINE | 31307 | 3511178 | 0.48 | 0.34 |
|  | **unclassified retroelement** | | 86294 | 19741473 | 2.69 | 1.9 |
| **Class II: DNA Transposon** | |  | 354328 | 119258669 | 16.25 | 11.46 |
|  | **TIR** |  |  |  |  |  |
|  |  | CMC | 60242 | 37800415 | 5.15 | 3.63 |
|  |  | hAT | 22861 | 5044257 | 0.69 | 0.48 |
|  |  | Mutator | 8807 | 7198249 | 0.98 | 0.69 |
|  |  | Tc1/Mariner | 22821 | 4529027 | 0.62 | 0.44 |
|  |  | PIF/Harbinger | 2349 | 1169488 | 0.16 | 0.11 |
|  |  | Other | 214427 | 58988206 | 8.04 | 5.67 |
|  | **Helitron** |  | 2738 | 1712469 | 0.23 | 0.16 |
| **Tandem Repeats** |  |  | 377777 | 56332911 | 7.68 | 5.42 |
| **Unkown** |  |  | 38393 | 12282923 | 1.67 | 1.18 |

**Table S10. Statistics of telomeric regions in** ***S. glauca* chromosomes.**

| Contig_Name | Contig_Size | Start_of_telo | End_of_telo | Size_of_telo | Telomeric_repeat_sequence | posi_of_telo | No_of_tel |
| --- | --- | --- | --- | --- | --- | --- | --- |
| Chr1A | 58306151 | 596680 | 614020 | 17340 | GGGTTTA | start | 2428.6 |
| Chr2A | 56542368 | 12375984 | 12407525 | 31541 | TTTAGGG | start | 4395.6 |
| Chr2B | 54134883 | 14737845 | 14768718 | 30873 | AACCCTA | start | 4310.3 |
| Chr3A | 58338586 | 41153013 | 41153976 | 963 | TTTAGGG | end | 135.6 |
| Chr3B | 54534240 | 23119400 | 23120362 | 962 | TTTAGGG | start | 135.6 |
| Chr5A | 52392194 | 50109135 | 50109744 | 609 | CTATACC | end | 91.4 |
| Chr7A | 52297465 | 45875782 | 45877190 | 1408 | ACCCTAA | end | 202 |
| Chr7B | 43274784 | 29550223 | 29551631 | 1408 | TTAGGGT | end | 202 |
| Chr8A | 38637212 | 27012 | 30638 | 3626 | AAACCCT | start | 508.3 |
| Chr8A | 38637212 | 6857589 | 6857849 | 260 | TTTAGGG | start | 37.4 |
| Chr8B | 36551220 | 32651350 | 32655348 | 3998 | AAACCCT | end | 563.3 |
| Chr9A | 32460120 | 324659 | 333667 | 9008 | TTTAGGA | start | 1267.3 |
| Chr9A | 32460120 | 324659 | 333667 | 9008 | TTTAGGATTTAGGG | start | 634.1 |
| Chr9A | 32460120 | 324676 | 333667 | 8991 | TTATGGTTCAGAGTTCAGGGT | start | 427 |
| Chr9B | 32186550 | 31990947 | 31999956 | 9009 | AATCCTA | end | 1263.3 |
| Chr9B | 32186550 | 31990947 | 31999956 | 9009 | AACCCTAAATCCTAAATCCTA | end | 423.1 |
| HAcontig5322 | 11705 | 1503 | 11705 | 10202 | TTAGGGT | end | 1463.7 |
| HBcontig172 | 2275 | 2 | 558 | 556 | AACCCTA | start | 80.1 |
| HBcontig195 | 41212 | 263 | 4053 | 3790 | AACCCTA | start | 531.7 |

**Table S11. ABCE genes in *S. glauca*.**

| Gene Catergories | Gene name | Haploptye A | Haploptye B |  |
| --- | --- | --- | --- | --- |
| A | *AGL13* | *Sg.2G0000924a* | *Sg.2G0000924b* |  |
|  | *AGL12* | *Sg.2G0002867a* | *Sg.2G0002867b* |  |
|  | *FUL-2* | *Sg.7G0000535a* | *Sg.7G0000535b* |  |
|  | *FUL* | *Sg.6G0002454a* |  | * |
|  | *AP1* | *Sg.4G0002225a* | *Sg.4G0002225b* |  |
| B | *PI* | *Sg.2G0001268a* | *Sg.2G0001268b* |  |
|  | *AP3* | *Sg.4G0002211a* | *Sg.4G0002211b* |  |
|  | *AGL32* | *Sg.9G0001514b* |  | * |
|  | *AGL32-2* | *Sg.1G0000893a* |  | * |
| C | *AG* | *Sg.5G0002405a* | *Sg.5G0002405b* |  |
| E | *SEP1* | *Sg.4G0002226a* | *Sg.4G0002226b* |  |
|  | *SEP1b* | *Sg.4G0002226p* |  | * |
|  | *SEP2* | *Sg.7G0000537a* | *Sg.7G0000537b* |  |
|  | *SEP3* | *Sg.4G0002679a* | *Sg.4G0002679b* |  |
