# Supplementary material for: Chromosome-scale genome sequence of Suaeda glauca sheds light on salt stress tolerance in halophytes
Source: Hortic Res. 2023 Aug 10;10(9):uhad161. doi: 10.1093/hr/uhad161 (PMC10506132; doi:10.1093/hr/uhad161)
Supplement: Web_Material_uhad161 [file web_material_uhad161.zip › 5.Supplementary results 1. The second whorls of S. glauca flower showed the sepal identity.docx]

**Supplementary results 1. The second whorls of *S. glauca* flower showed the sepal identity**

1. **Sepal-like identity of the second whorl in S. glauca flower**

To confirm the sepal-like identity of the second whorl in *Suaeda glauca* flowers, we analyzed the expression of genes involved in photosynthesis and chlorophyll synthesis-related pathways (ath00195, ath00196, ath00710, and ath00860) (Figure S1-S4). The clustered heatmaps showed that the expression patterns of the examined genes in the second whorl were similar to those in the first whorl.


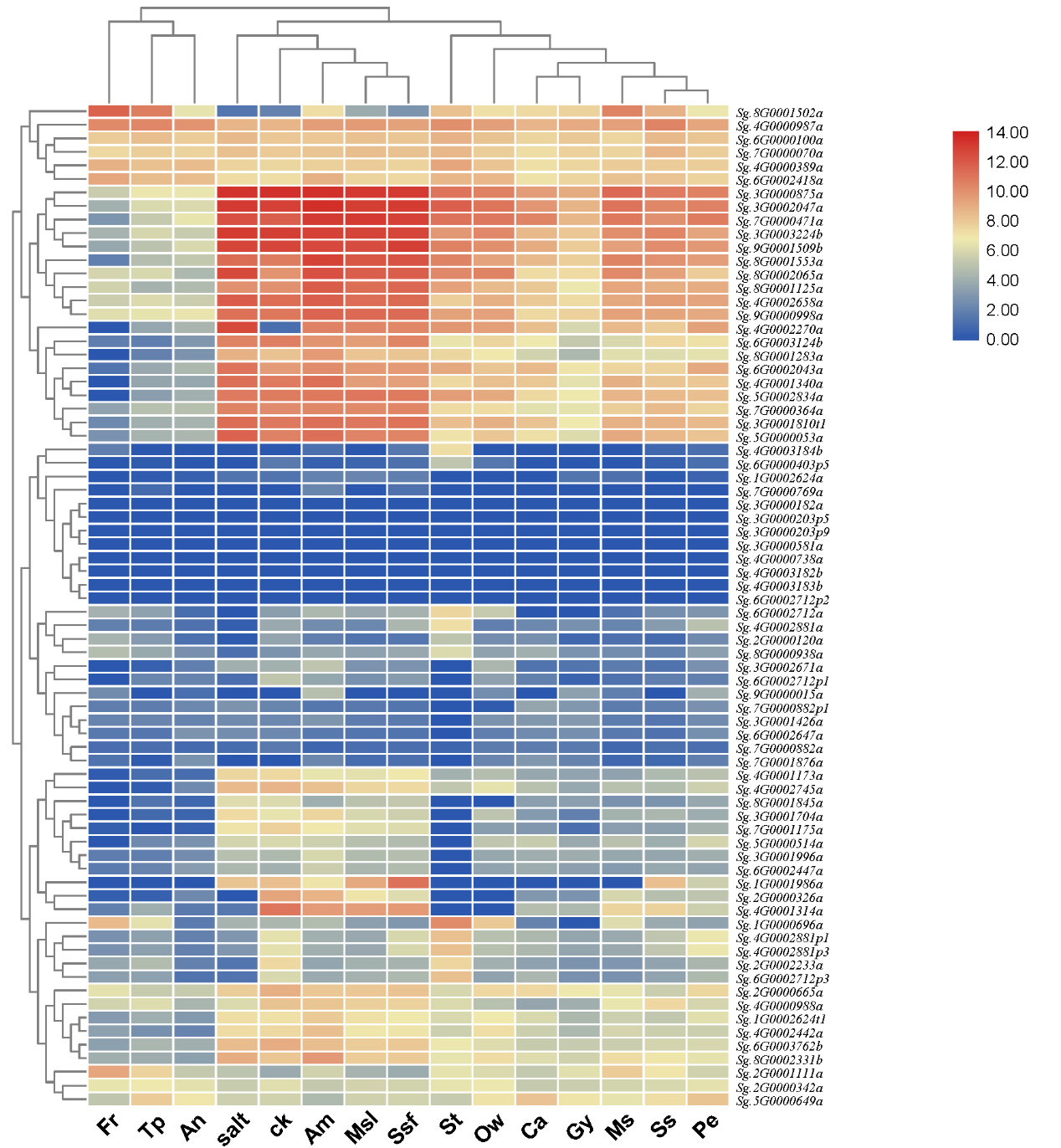


**Figure S1. Expression levels of photosynthesis genes in different tissues of *Suaeda glauca*.** The Arabidopsis genes involved in the ath00195 pathway (Photosynthesis - Arabidopsis thaliana (thale cress); <https://www.genome.jp/dbget-bin/www_bget?pathway:ath00195>) were extracted from the KEGG website, and their homologs in *Suaeda glauca* were identified using BLASTP. The expression levels of these genes are shown in the heatmap, and the samples and genes are clustered based on the expression data.


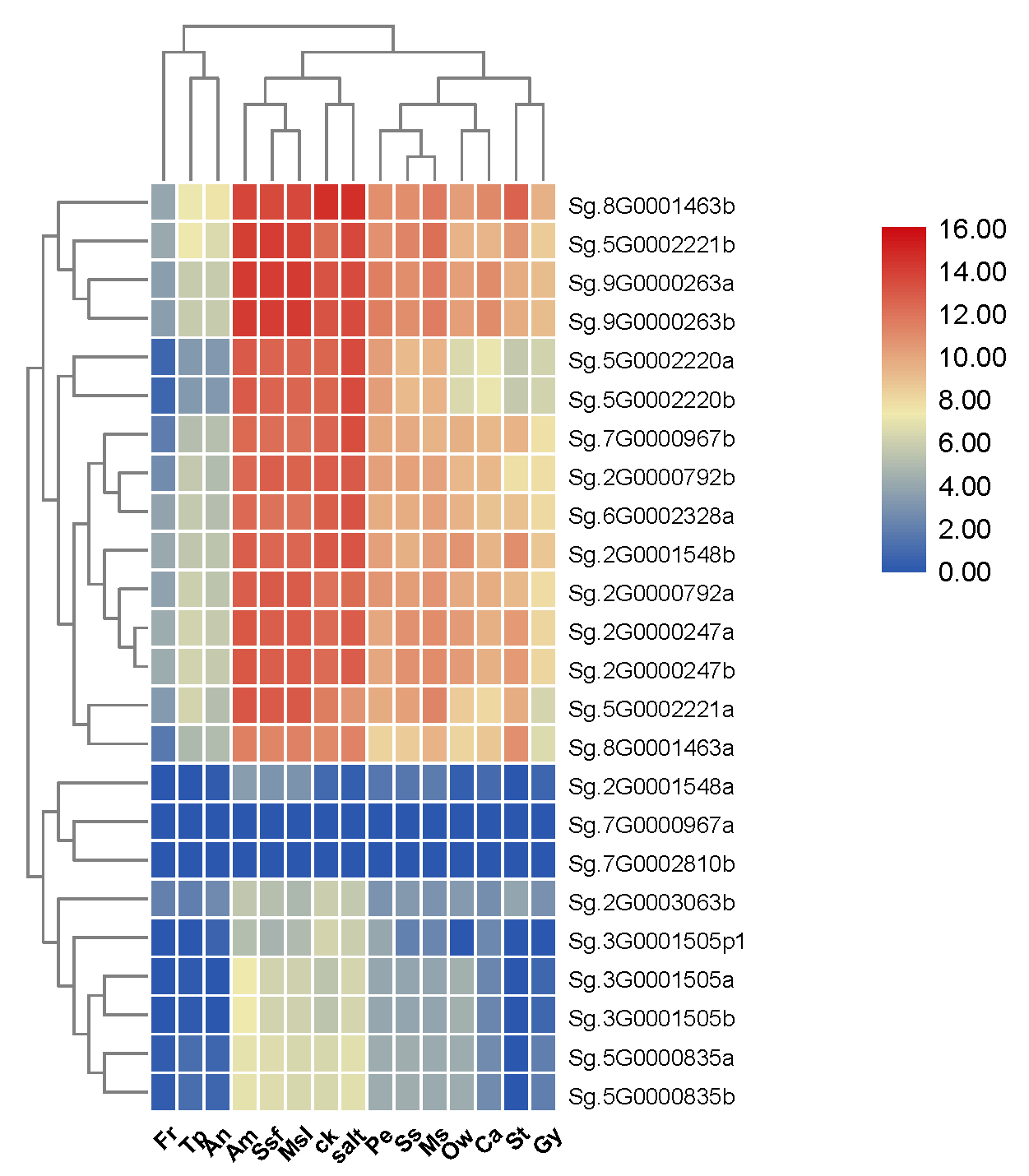


**Figure S2. Expression of antenna protein-coding genes in various tissues of *Suaeda glauca*.** The Arabidopsis genes on the ath00196 pathway (Photosynthesis - antenna proteins - Arabidopsis thaliana (thale cress); <https://www.genome.jp/dbget-bin/www_bget?pathway:ath00196>) were extracted from the KEGG website, and their homologs in *Suaeda glauca* were identified based on the blastp results. The expression levels of these genes are shown in the heatmap, and the samples and genes are clustered based on the expression data.


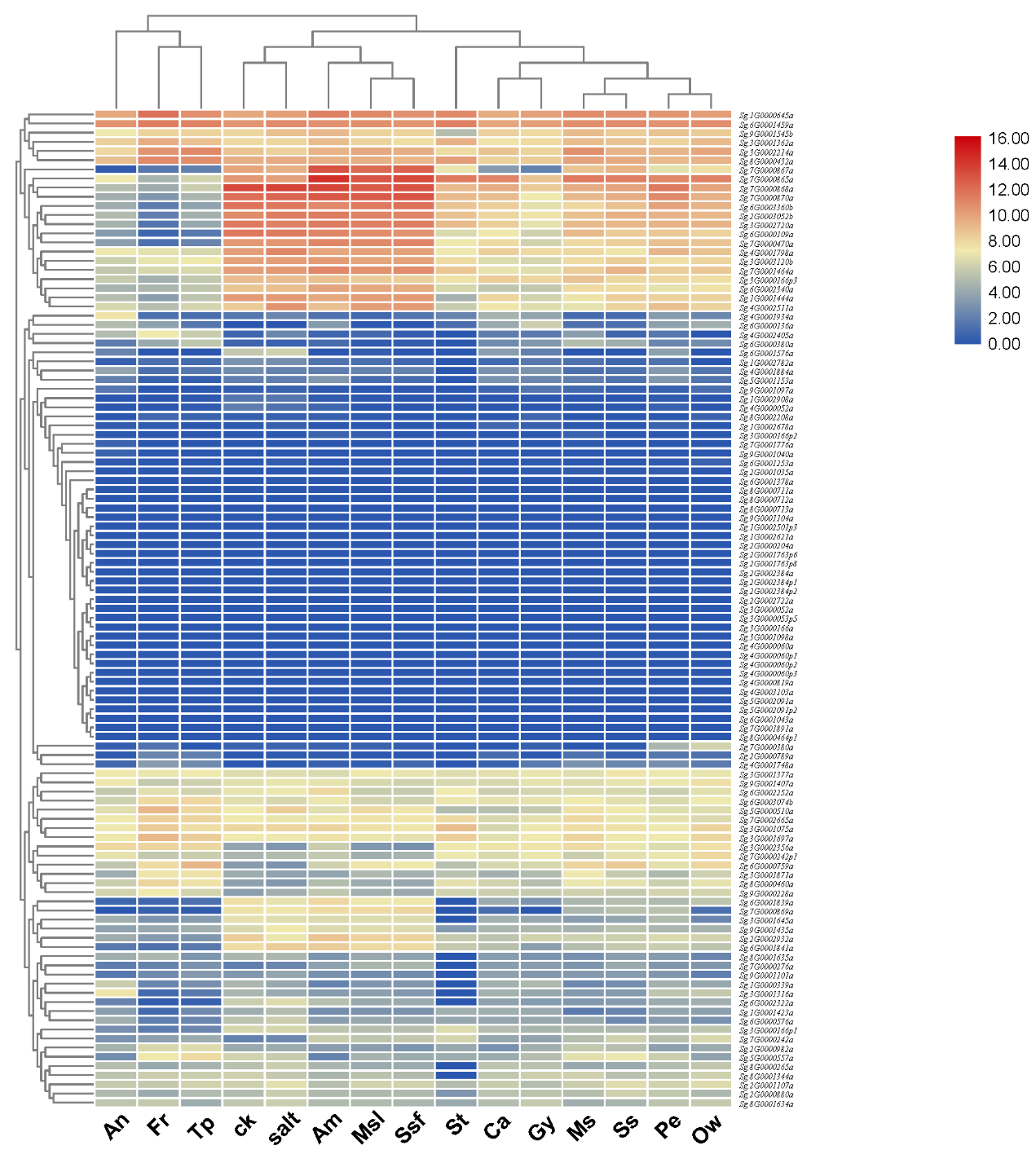


**Figure S3. Expression levels of carbon fixation-related genes in different tissues of *Suaeda glauca*.** The Arabidopsis genes involved in the ath00710 pathway (carbon fixation in photosynthetic organisms - Arabidopsis thaliana (thale cress); <https://www.genome.jp/dbget-bin/www_bget?pathway:ath00710>) were extracted from the KEGG website and homologs in *Suaeda glauca* were identified based on the balstp results. The heatmap shows the expression levels of the identified genes across different tissues. Samples and genes are clustered based on expression data.


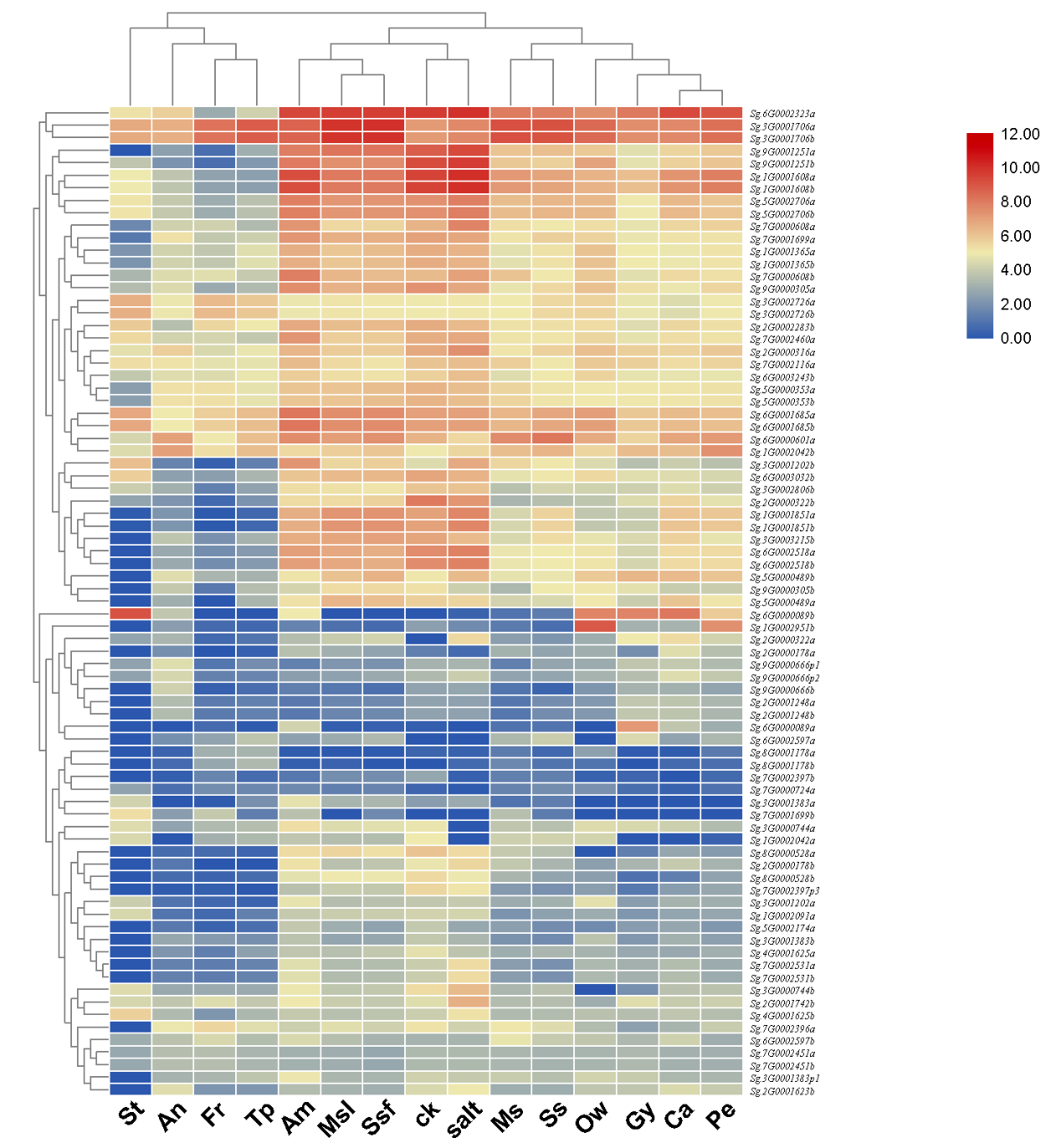


**Figure S4. Expression of porphyrin and chlorophyll metabolism genes in different tissues of *Suaeda glauca*.** Arabidopsis genes on the ath00860 pathway (Porphyrin and chlorophyll metabolism - Arabidopsis thaliana (thale cress); <https://www.genome.jp/dbget-bin/www_bget?pathway:ath00860>) were extracted from KEGG website, and homologs in *Suaeda glauca* were identified based on blastp results. Expression levels are presented in the heatmap above, and samples and genes are clustered based on the expression data. *Sg6G0002323a* showed high expression levels in all green tissues, and its ortholog in Arabidopsis is *AT1G74470*.

**2.** **The expression and function of AT1G74470 in Arabidopsis**

The ortholog of Sg6G0002323 in Arabidopsis is AT1G74470 (<https://www.arabidopsis.org/servlets/TairObject?id=28505&type=locus>). It encodes a multifunctional protein with geranylgeranyl reductase activity that has been shown to catalyze the reduction of prenylated geranylgeranyl-chlorophyll a to phytyl-chlorophyll a (chlorophyll a) and free geranylgeranyl pyrophosphate to phytyl pyrophosphate. The gene is expressed in photosynthetic tissues, and its function is essential for chlorophyll synthesis and photosynthesis in Arabidopsis (Figure S5-S7).


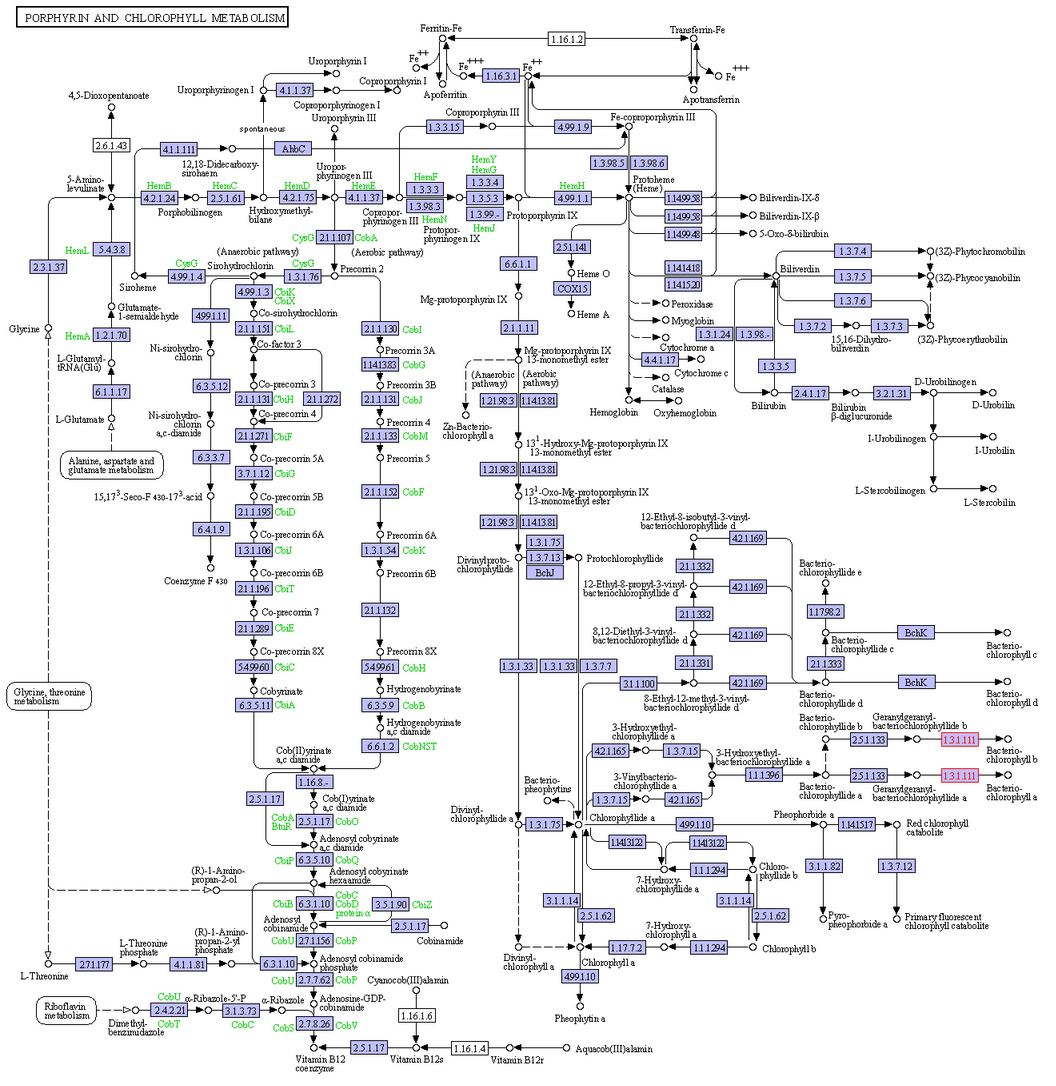


**Figure S5. The protein encoded by AT1G74470 catalyzes the last step of chlorophyll synthesis.** The protein encoded by AT1G74470 catalyzes the final step of chlorophyll synthesis by converting prenylated geranylgeranyl-chlorophyll a to phytyl-chlorophyll a and free geranylgeranyl pyrophosphate to phytyl pyrophosphate.


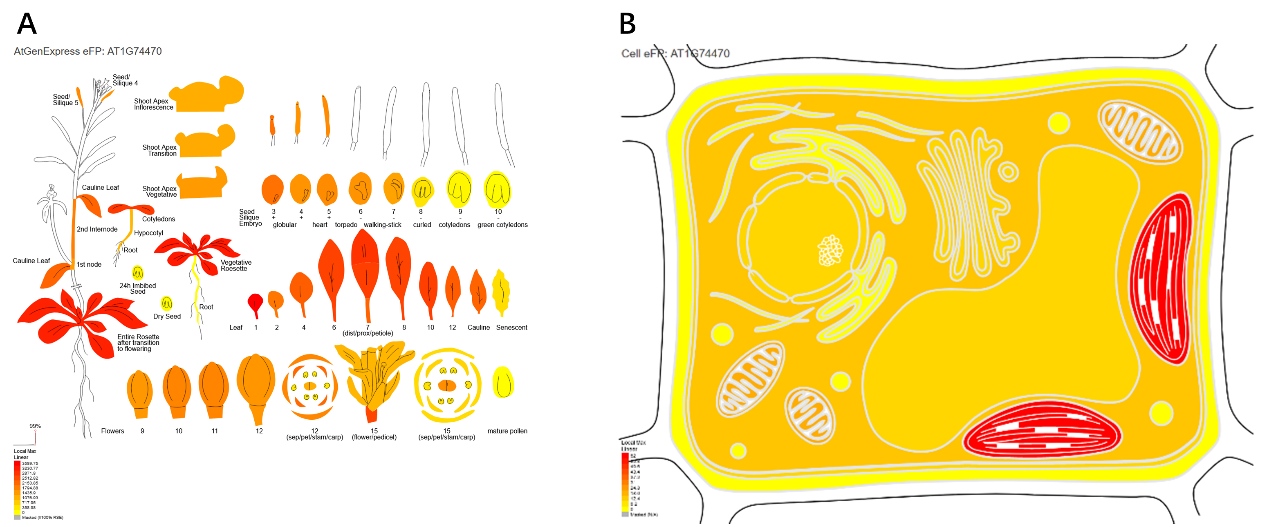


**Figure S6. AT1G74470 is expressed in photosynthetic tissues and organelles.** (A) The expression pattern of AT1G74470 in various tissues. (B) The subcellular expression pattern of AT1G74470. The data are from ePlant database (<http://bar.utoronto.ca/eplant/>).


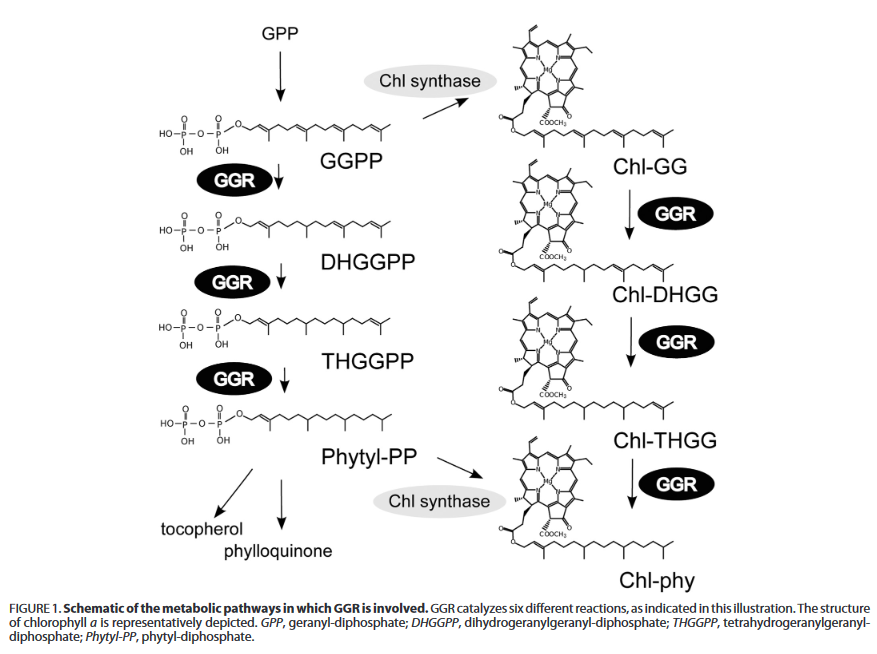


**Figure S7.** **The protein encoded by AT1G74470 catalyzes multiple reactions involved in chlorophyll synthesis, including the last step.** This illustration depicts the chemical structures of the intermediates and products of the reactions catalyzed by the enzyme. GPP, geranyl-diphosphate; DHGGPP, dihydrogeranylgeranyl-diphosphate; THGGPP, tetrahydrogeranylgeranyl-diphosphate; Phytyl-PP, phytyl-diphosphate (adapted from Kaori et al., 2013).
